# Highly efficient magnetic labelling allows MRI tracking of the homing of stem cell‐derived extracellular vesicles following systemic delivery

**DOI:** 10.1002/jev2.12054

**Published:** 2021-01-15

**Authors:** Zheng Han, Senquan Liu, Yigang Pei, Zheng Ding, Yuguo Li, Xinge Wang, Daqian Zhan, Shuli Xia, Tom Driedonks, Kenneth W. Witwer, Robert G. Weiss, Peter C.M. van Zijl, Jeff W.M. Bulte, Linzhao Cheng, Guanshu Liu

**Affiliations:** ^1^ Russell H. Morgan Department of Radiology Johns Hopkins University School of Medicine Baltimore Maryland USA; ^2^ F.M. Kirby Research Center Kennedy Krieger Institute Baltimore Maryland USA; ^3^ Cellular Imaging Section and Vascular Biology Program Institute for Cell Engineering Johns Hopkins University School of Medicine Baltimore Maryland USA; ^4^ Department of Medicine Johns Hopkins University School of Medicine Baltimore Maryland USA; ^5^ Division of Life Sciences and Medicine University of Science and Technology of China Hefei Anhui China; ^6^ Department of Radiology Xiangya Hospital Central South University Changsha Hunan China; ^7^ Department of Bioengineering University of Illinois at Chicago Chicago Illinois USA; ^8^ Department of Neurology Hugo W. Moser Research Institute at Kennedy Krieger Baltimore Maryland USA; ^9^ Department of Molecular and Comparative Pathobiology Johns Hopkins University School of Medicine Baltimore Maryland USA; ^10^ Division of Cardiology Department of Medicine Johns Hopkins University School of Medicine Baltimore Maryland USA

**Keywords:** acute kidney injury, extracellular vesicle, iPSC, MRI, myocardial injury, stem cell

## Abstract

Human stem‐cell‐derived extracellular vesicles (EVs) are currently being investigated for cell‐free therapy in regenerative medicine applications, but the lack of noninvasive imaging methods to track EV homing and uptake in injured tissues has limited the refinement and optimization of the approach. Here, we developed a new labelling strategy to prepare magnetic EVs (magneto‐EVs) allowing sensitive yet specific MRI tracking of systemically injected therapeutic EVs. This new labelling strategy relies on the use of ‘sticky’ magnetic particles, namely superparamagnetic iron oxide (SPIO) nanoparticles coated with polyhistidine tags, to efficiently separate magneto‐EVs from unencapsulated SPIO particles. Using this method, we prepared pluripotent stem cell (iPSC)‐derived magneto‐EVs and subsequently used MRI to track their homing in different animal models of kidney injury and myocardial ischemia. Our results showed that iPSC‐derived EVs preferentially accumulated in the injury sites and conferred substantial protection. Our study paves a new pathway for preparing highly purified magnetic EVs and tracking them using MRI towards optimized, systemically administered EV‐based cell‐free therapies.

## 1 INTRODUCTION

Extracellular vesicles (EVs) are small membranous blebs or vesicles released from nearly all cell types that function as important messengers and mediators for intercellular communication in a diverse range of biological processes (Hoshino et al., 2015; Karpman et al., 2017). Depending on their biogenesis (Van Niel et al., 2018), EVs are typically classified into exosomes (50–150 nm in diameter) or microvesicles (50–500 nm in diameter). Exosomes are derived from specialized intracellular compartments, that is endosomes or multi‐vesicular bodies (MVBs), while microvesicles are shed directly from the plasma membrane. When reaching recipient cells that are either in the immediate vicinity or at a distance, EVs can be internalized by recipient cells to transmit cargo (e.g. proteins, messenger RNA, microRNA and lipids) and elicit biologic changes in recipient cells (Colombo et al., 2014). In stem cell research, increasing evidence shows that EVs are essential for stem cells to protect or regenerate injured cells, presumably through an EV‐mediated paracrine effect (Chen et al., 2008; Gnecchi et al., 2008). This has led to the use of stem cell‐derived EVs alone for cell‐free therapy (Marbán, 2018; Rani et al., 2015). Indeed, compared to parental mesenchymal stem cells (MSCs) and induced pluripotent stem cells (iPSCs), human stem cell‐derived EVs are considered to be a safer and more effective regenerative medicine approach for treating many otherwise untreatable diseases such as kidney injury (Bruno et al., 2009, 2012; Camussi et al., 2012; Collino et al., 2015) and cardiac disease (Adamiak et al., 2018; Liu et al., 2018). Despite the appeal and potential impact of EV‐based therapies, there are currently no clinic‐ready methods for noninvasively tracking them in vivo to guide, optimize, and monitor treatment.

Noninvasive in vivo imaging approaches are needed for tracking the delivery, uptake and fate of administered EVs over time. Importantly, such methods could be used to assess the quantity of administered EVs in the organs to be treated, which may be predictive of therapeutic responses. Before this, EV imaging and tracking methods can serve as a guide to direct the development, optimization, and implementation of EV‐based therapeutics. To date, considerable effort has been devoted to developing EV tracking methods using fluorescence imaging (Antes et al., 2018; Grange et al., 2014; Wiklander et al., 2015), bioluminescence imaging (Imai et al., 2015), single‐photon emission computed tomography (SPECT) (Hwang et al., 2015), positron emission tomography (PET) (Shi et al., 2019), computed tomography (CT) (Betzer et al., 2017; Perets et al., 2019), magnetic resonance imaging (MRI) (Busato et al., 2016; Hu et al., 2015) and magnetic particle imaging (Jung et al., 2018). Among them, MRI is an appealing imaging modality because it is used widely in the clinic and has excellent soft‐tissue contrast without using ionizing radiation. To the best of our knowledge, there are only two reports on in vivo MRI‐based EV‐tracking studies. In the study reported by Busato et al. (2016), superparamagnetic iron oxide (SPIO) particles were introduced into the parental cells (adipose stem cells) and the EVs secreted from these cells were then collected. While this approach does not require a purification procedure to remove free SPIO, the labelling efficiency was unfortunately found to be low, making in vivo tracking of EVs challenging due to the inherently low sensitivity of MRI. Hu et al. used electroporation to load SPIOs into EVs and subsequently purified them by ultracentrifugation (Hu et al., 2015). While this approach provides high labelling, ultracentrifugation induces SPIO aggregation when pelleting and is not an efficient method for removing free naked SPIO nanoparticles. Indeed, all previous MRI detection approaches were demonstrated on locally injected EVs and, to the best of our knowledge, no MRI tracking of systemically administered EVs has been reported. Hence, an efficient technology to obtain highly purified SPIO‐labelled EVs that can be tracked to injured tissues following systemic administration is still an unmet need.

The aim of this study was to first develop a platform technology for preparing highly purified magnetically labelled EVs (magneto‐EVs) and then to test in vivo MRI tracking of their tissue‐specific uptake and targeted delivery in two disease sites, that is kidney and heart. We chose to use iPSC‐derived EVs because iPSCs are more amenable to generating EVs which could protect or repair injury, as demonstrated in preclinical studies (Adamiak et al., 2018; Lee et al., 2012). We have recently shown that human iPSCs can be cultured infinitely in chemically defined medium (free of exogenous EVs from serum or other biological sources) and produce EVs markedly higher than other types of stem cells (Liu et al., 2019). Considering that stem cell derived EVs lack nuclei, iPSC‐derived EVs are attractive without the concerns of genetic instability and tumorigenicity associated with the administration of whole iPSCs (Jung et al., 2017; Shi et al., 2017). Moreover, individualized autologous iPSCs can be generated from a single blood draw or skin sample from the same patient to minimize immunogenicity and ethical concerns (Jung et al., 2017). Using different animal tissue injury models, we show here that the temporal‐spatial distribution of systemically‐injected, magnetically labelled, iPSC‐derived EVs can be assessed quantitatively in vivo with MRI.

## 2 MATERIALS AND METHODS

### 2.1 Materials

Carboxyl SPIOs (SPIO‐COOH, core diameter = 5 nm) was purchased from Ocean Nanotech (Springdale, AR). Hexa‐histidine peptide was purchased from GeneScript (Springdale, AR, USA). 1‐Ethyl‐3‐(3‐dimethylaminopropyl)‐carbodiimide (EDC), sulfo‐N‐hydroxysuccinimide (sulfo‐NHS) and Ni‐NTA were purchased from Sigma‐Aldrich (St. Louis, MO, USA).

### 2.2 Synthesis of SPIO‐His

Fifty microliters of SPIO‐carboxyl (2 mg ml^‐1^) was mixed with 100 μg EDC (10 mg ml^‐1^) and 100 μg NHS (10 mg ml^‐1^), and 100 μl MES buffer (pH 6.0). The solution was shaken for 30 min. One mg of peptide (25 mg ml^‐1^ in dd H_2_O) was then added, followed by adding 200 μl 10 mM PBS, pH 7.4. The pH of the solution was then adjusted to 7.4, followed by shaking for 2 h. The product was dialyzed, lyophilized and reconstituted to 2 mg Fe per ml.

### 2.3 Cell culture

Human iPSCs were cultured in 6‐well plates coated with vitronectin (Gibco, Calsbad, CA, USA) using the chemically defined Essential 8 (E8) medium (Gibco) at 37°C in a humidified atmosphere of 5% CO_2_ and 95% air as previously described (Chou et al., 2011). The culture medium was changed daily after gently washing the cells with 10 mM PBS, pH 7.4. Cells were passaged at 80%–90% (approximately 2 × 10^6^ cells) confluence using TrypLE Express Enzyme (Gibco) supplemented with 10 μM Y‐27632 dihydrochloride. Cells were routinely checked for mycoplasma contamination.

### 2.4 Collection, purification and enrichment of EVs

A total of 300 ml medium was harvested from of iPSC culture according to the protocol reported previously (Liu et al., 2019). Human iPSCs with a normal karyotype and a passage number of 60–80 were used. Conditioned culture medium was centrifuged for 10 min at 300 × *g* followed by another 10 min at 2000 × *g* at 4 °C to remove cells and debris, and concentrated using an Amicon ultra‐15 filter column and an Ultracel‐100 membrane by centrifugation at 4000 × *g* for 20 min (MilliporeSigma, Billerica, MA, USA). Each time, 15 ml medium was loaded to the column and retentate (approximately 400 μl) was collected. Then, the concentrated EVs solution was purified by size exclusion chromatography (SEC) using qEV columns (iZON, Cambridge, MA, USA). Briefly, after rinsing the qEV columns with PBS, 0.5 ml fraction of the concentrated EVs were applied to the top of the columns and eluted with PBS. Three EV‐rich fractions (7–9, 0.5 ml each) were pooled. The purified EVs were further concentrated using an Amicon column and final volume was adjusted so that the final concentration is about 1 × 10^11^ EVs per ml. The size of EVs was measured by dynamic light scattering (DLS, Nanosizer ZS90, Malvern Instruments), and the numbers of EVs were measured using a nanoparticle tracking analysis (NTA) instrument (Zetaview, Particle Metrix, Germany) using a 488‐nm laser and ZetaView 8.04.02 software. The protein content of EV was measured using a Micro BCA Protein Assay Kit (Thermo Fisher Scientific, Waltham, MA, USA).

### 2.5 Electroporation of EVs

Fifty microliters of concentrated EVs (1.1 × 10^11^ per ml) were mixed with 25 μl of 2 mg ml^‐1^ SPIO‐His. Electroporation was performed with a Gene Pulser Xcell Electroporation Systems (Biorad) using two 5s‐pulses of 240 V mm^‐1^ and 100 F Capacitance with a 1 mm cuvette. EVs were then transferred to a clean microcentrifuge tube and placed on ice for 1 h before Ni‐NTA purification.

### 2.6 Purification of magneto‐EVs

Ni‐NTA columns were prepared by packing 1 ml Ni‐NTA His•Bind resins (Sigma) into a 6‐ml ISOLUTE Single Fritted Reservoir column with 10 μm polyethylene frit (Biotage, Charlotte, NC, USA), followed by washing with 5 ml PBS. After electroporation, EVs (∼75 μl) were resuspended in 200 μl PBS and then loaded onto a Ni‐NTA column and gently shaken for 15 min. After the first elute was collected, the column was rinsed with 200 μl PBS. All elutes were collected. A total of three times of Ni‐NTA filtrations were performed to minimize the residual non‐encapsulated SPIOs in the final EV solution. To further enrich magneto‐EVs, the microcentrifuge tube containing purified magneto‐EVs was fixed upright on a 1‐inch cube Neodymium magnet (CMS magnetics, Garland, TX, USA) overnight and the pelleted magneto‐EVs were resuspended in the desirable volume of PBS. Isolation and purification of fetal bovine serum (FBS) (catalog #F2442, Sigma‐Aldrich) derived EVs were performed in a similar manner.

To test the ability to enrich EVs using magnetic field, 200 μl magneto‐EVs were transferred to a 1 ml centrifuge tube and an external magnetic field (∼0.66 Tesla) was applied using a N52 grade neodymium magnet cube (1 inch, CMS magnetics, Plano, TX, USA) for overnight.

The iron content of electroporated EVs was calculated using the equation: iron (mg ml^‐1^) = [(OD500‐OD800) × dilution factor]/4.3, where OD is the optical density at the wavelength of 500 and 800 nm respectively, and 4.3 is the extinction coefficient for SPIO (Hu et al., 2015). The size of EVs was measured by DLS, and the loss of EVs during the purification procedure, the numbers of EVs before and after purification were measured using NTA.

### 2.7 Preparation of iPSC‐EVs using the conventional method

To compare the labelling efficiency of our approach with that of conventional method, we also prepared SPIO‐labelled EVs using the previously reported protocol (Busato et al., 2016). We incubated iPSC with SPIO‐COOH in culture medium (final iron concentration = 200 μg ml^‐1^) for 24 h. Then the cells were washed with PBS and fresh medium was added. After incubating for another 48 h, EVs in the cell culture medium were harvested and purified using the aforementioned protocols.

### 2.8 In vitro MRI characterization

Samples of SPIO‐COOH, SPIO‐His, unlabelled and labelled EVs were prepared at different concentrations in 10 mM PBS, pH 7.4 and transferred to 5 mm glass NMR tubes, and then combined for MRI measurements on a Bruker 9.4 Tesla vertical bore scanner equipped with a 20 mm birdcage transmit/receive coil. T_2_ relaxation times were measured using the Carr‐Purcell‐Meiboom‐Gill (CPMG) method at room temperature as previously described (Zhang et al., 2018). The acquisition parameters were: TR/TE = 25 s/4.3 ms, RARE factor = 16, matrix size = 64 × 64, in‐plane resolution = 0.25 × 0.25 mm and slice thickness = 2 mm. Each T_2_w image took approximately 1′40″ to acquire. The T_2_ relaxivities (r_2_) were calculated based on mean R_2_ ( = 1/T_2_) of each sample and their concentrations (C), using the following equation:

$$R_{2}=R_{2}^{0}+r_{2}C$$

where R_2_^0^ represents the inherent water proton transverse relaxation rate.

### 2.9 Determination of efficiency of Ni‐NTA column to remove unencapsulated SPIO‐His

Seventy‐five microliters of SPIO‐His solution (0.67 mg ml^‐1^) was diluted with 200 μl PBS and loaded into 1 ml Ni‐NTA His•Bind resins (Sigma). After gently shaking for 15 min, the eluate was collected. The column was washed using 200 μl PBS, and the eluate was collected again. Collected eluates were mixed in an Amicon ultra‐0.5 filter column with an Ultracel‐3 membrane (MilliporeSigma, Billerica, MA, USA) and concentrated to 75 μl using ultrafiltration at 4000 × *g* for 20 min.

The R_2_ value of the concentrated eluate was measured using the MRI method described above, and the concentration of SPIO‐His (C_eluate_) was calculated using a predetermined standard curve plotted by the R_2_ values of five SPIO‐His solutions (concentration = 0.42 to 6.7 μg m^‐1^). The efficiency of the column in removing unencapsulated SPIO‐His was calculated as C_eluate_ x V_eluate_/(C_initial_ xV_initial_)× 100%, where C_initial _= 0.67 mg ml^‐1^ and V_initial _= 75 μl.

### 2.10 EV labelling efficiency

SPIO‐labelled EVs (50 μl, ∼1.1 × 10^11^ EV per ml) prepared by different methods were transferred to a microcentrifuge tube and the same Neodymium magnet as described previously was used to separate magneto‐EVs from EVs without SPIO labelling. Twenty‐four hours later, the supernatant was collected. The numbers of EVs were measured using nanoparticle tracking analysis (NTA), and the labelling efficiency was calculated as: labelling efficiency (%) = (1 – N_supernantant_ /N_total_) x 100%.

### 2.11 Transmission electron tomography

Carbon‐coated 400 mesh copper grids (Electron Microscopy Services, Hatfield, PA, USA) were placed on 30 μl drops of SPIO or EV samples for 1 min. Grids were quickly washed with two drops of dH_2_O and blotted dry on a filter paper. Grids were stained for one minute with 2% uranyl acetate (Electron Microscopy Services) in dH_2_O and blotted dry. All imaging was performed with a Zeiss Libra 120 TEM operated at 120 KV and equipped with an Olympus Veleta camera (Olympus Soft Imaging Solutions GmbH, Münster, Germany).

### 2.12 Mouse models of acute kidney injury

All animal experiments were approved by our Animal Care and Use Committee. Male C57BL/6J mice (6–8 weeks of age) were acquired from Jackson Laboratories (Bar Harbor, ME, USA). The LPS‐AKI model was established as described previously (Liu et al., 2018) by intraperitoneal (i.p.) administration of lipopolysaccharide (LPS, Sigma Aldrich) at a dose of 10 mg kg^‐1^. The IRI‐AKI model was prepared according to a previously published procedure (Wei & Dong, 2012). In brief, animals were anesthetized with 2% isoflurane and an incision was made in the back muscle and skin layer to expose the right kidney and the renal vascular pedicle was clamped using a microvessel clamp (#18052‐03, Fine Science Tools, Foster City, CA, USA) for 45 min, followed by suture‐closing the incisions.

### 2.13 Mouse model of heart ischemic and reperfusion injury (IRI)

The cardiac IRI protocol was performed as previously described (Naumova et al., 2006). In brief, male C57BL/6J mice (*n* = 3) were induced anaesthesia using 3%–4% isoflurane and 0.03–0.07 mg kg^‐1^ buprenorphine (subcutaneously, s.c.). Then, anaesthesia was maintained by 1%–2% isoflurane and with 2 mg kg^‐1^ succinylcholine (i.p.). Mice were intubated and ventilated with a MiniVent ventilator (Harvard Apparatus, Holliston, MA, USA) (Naumova et al., 2006; Yap et al., 2019), and the body temperature was maintained constant as monitored with a rectal probe. After 5 min, a left thoracotomy was performed in the 5th to 6th intercostal space. The pericardium was torn and the coronary artery was located. A piece of 7‐0 prolene was tied around the coronary artery with a small piece of black polyethylene PE10 tubing (Braintree Scientific Inc., Braintree, MA, USA) under the suture. Occlusion was performed for 35 min. During occlusion, ribs were closed with one single 5‐0 silk suture and the skin was closed with a bulldog clamp (Fine Science Tools, Foster City, CA, USA). At 5 min prior to ending the occlusion, the chest was reopened carefully and the sutures were removed. Ribs and skin were closed with 5‐0 silk. Mice were allowed to regain consciousness and a second dose of buprenorphine at the dose of 0.06–0.075 mg kg^‐1^ was administered s.c.

### 2.14 In vivo MRI

All animal studies were performed on a 11.7T Biospec (Bruker) horizontal bore scanner equipped with a mouse brain surface array RF coil (receiver) and a 72 mm volume coil (transmitter). A 30‐min dynamic scan was acquired using a fast low angle shot (FLASH) gradient echo sequence immediately before i.v. injection of 1 × 10^9^ magneto‐EVs or 10 ng SPIO‐His (having the same iron amount as that in magneto‐EVs) in 200 μl PBS. The acquisition parameters were: flip angle = 25°, TR = 800 ms, TE = 5.8 ms, matrix size = 256 × 128 and resolution = 167 × 280 mm^2^. Before and 30 min after injection T_2_* maps were also acquired using a multiple gradient echo (MGE) pulse sequence with TR = 800 ms and TE times of 2.6, 5.8, 9, 12.2, 15.4, 18.6, 21.8, and 25 ms.

After MRI, mice were euthanized by cervical dislocation under anaesthesia and tissues of interest were collected and fixed in 4% paraformaldehyde solution for ex vivo MRI and histological analysis.

### 2.15 Ex vivo MRI

Excised kidneys and hearts were transferred to a 5 ml syringe filled with proton‐free fluid Fomblin (Solvay Solexis, Inc., USA) and ex vivo high‐resolution MRI was performed on a vertical bore 9.4T Bruker scanner equipped with a 15 mm birdcage transmit/receive volume coil. A three‐dimensional FLASH sequence was used with TE = 6 ms, TR = 150 ms, matrix size = 310 × 240 × 145, FOV = 12.06 × 9.48 × 5.68 mm, resolution = 39 × 39 × 39 μm^3^, averages = 5, and flip angle = 15°. The total scan time was 6h43m12s. Amira 3D Visualization Software 5.4.3 (Visage Imaging Inc., Carlsbad, CA, USA) was used to quantify the areas of hypointense signal and to visualize the 3D distribution of magneto‐EVs.

### 2.16 Fluorescence imaging

Forty microliters of iPSC‐EV (1.1 × 10^11^ per ml) were incubated with DiR dye (1,1′‐dioctadecyl‐3,3,3′,3′‐Tetramethylindotricarbocyanine Iodide, ThermoFisher Scientific) at the final concentration of 1 μM for 15 min with gentle shaking, followed by eluting through a Sephadex G‐50 column to remove free DiR dyes. Normal control (*n* = 4) and LPS‐AKI mice (*n* = 4) were i.v. injected with DiR‐iPSC‐EV (1 × 10^9^ in 200 μl PBS) and euthanized at 30 min after injection to collect organs. Fluorescence imaging was performed using the Spectrum/CT IVIS in vivo imaging system using the Ex720/Em790 filter set and analyzed using the Living Image software (PerkinElmer, Waltham, MA) .

### 2.17 Histological analysis

Excised tissues were paraffin‐embedded and sectioned at 5 μm thickness and stained with Prussian blue and Periodic acid–Schiff (PAS). Sections were imaged using a Zeiss Axio Observer Z1 microscope (Zeiss, Oberkochen, Germany) and processed using Zen Pro software.

### 2.18 Immunofluorescence staining

Mouse kidney slices from formalin‐fixed and paraffin‐embedded tissue blocks underwent deparaffinization and rehydration using standard protocols. The slices were then treated with cold acetone for 10 min at ‐20 °C and blocked with 1% BSA for 1 h at room temperature (RT). For VCAM‐1 staining, the FITC‐conjugated anti‐VCAM‐1 antibodies (sc‐18864 FITC, Santa Cruz Biotechnology, Santa Cruz, CA, USA; 1:50 dilution in 1% BSA) were applied to the slides and incubated at RT for 1 h at dark. Nucleus were stained with DAPI. Cover glass was mounted using the Prolong Antifade Mountant (Thermo Fisher Scientific). The fluorescence images of tissues were then acquired using a Zeiss Apotome microscope (Zeiss, Oberkoche, Germany).

### 2.19 Treatment effects of iPSC‐EV on LPS‐induced AKI mice

Eight randomly selected mice were administered with iPSC‐EV (2 × 10^9^ EVs in 200 μl PBS) or vehicle control (200 μl PBS) intravenously through the tail vein at the same time as LPS injection. Animal survival was monitored for each group every day up to 6 days. Blood was drawn from the tail vein at 24 h after LPS injection and serum creatinine (SCr) level was measured using a creatinine detecting kit (Sigma, St Louis, MO, USA).

### 2.20 Proteomic analysis

Protein samples from iPSC‐EV and human plasma EVs were acquired and analysed using a protocol reported previously (Liu et al., 2019). Briefly, digested protein samples underwent liquid chromatography–mass spectrometry (LC‐MS)/MS analysis and quantification using the Intensity Based Absolute Quantification (iBAQ) algorithm in MaxQuant. A stringent log fold change threshold (|logFC | > = 5) was used to define if a protein is significantly changed between iPSC‐EV and human plasma EVs. Gene ontology enrichment analysis was performed using the ‘clusterProfiler’ R package from Bioconductor, using species humans as the annotation database. Upregulated genes were clustered using the biological process classification.

### 2.21 Statistical analysis

All data are presented as mean ± SEM unless otherwise noted. GraphPad Prism version 8 (GraphPad Software Inc., San Diego, CA, USA) was used to perform statistical analysis. An unpaired two‐tailed Student's t‐test was used to compare the difference between two groups. Differences with *P *< 0.05 were considered statistically significant. The Kaplan‐Meier method was used to analyse animal survival data.

## 3 RESULTS

**FIGURE 1:**
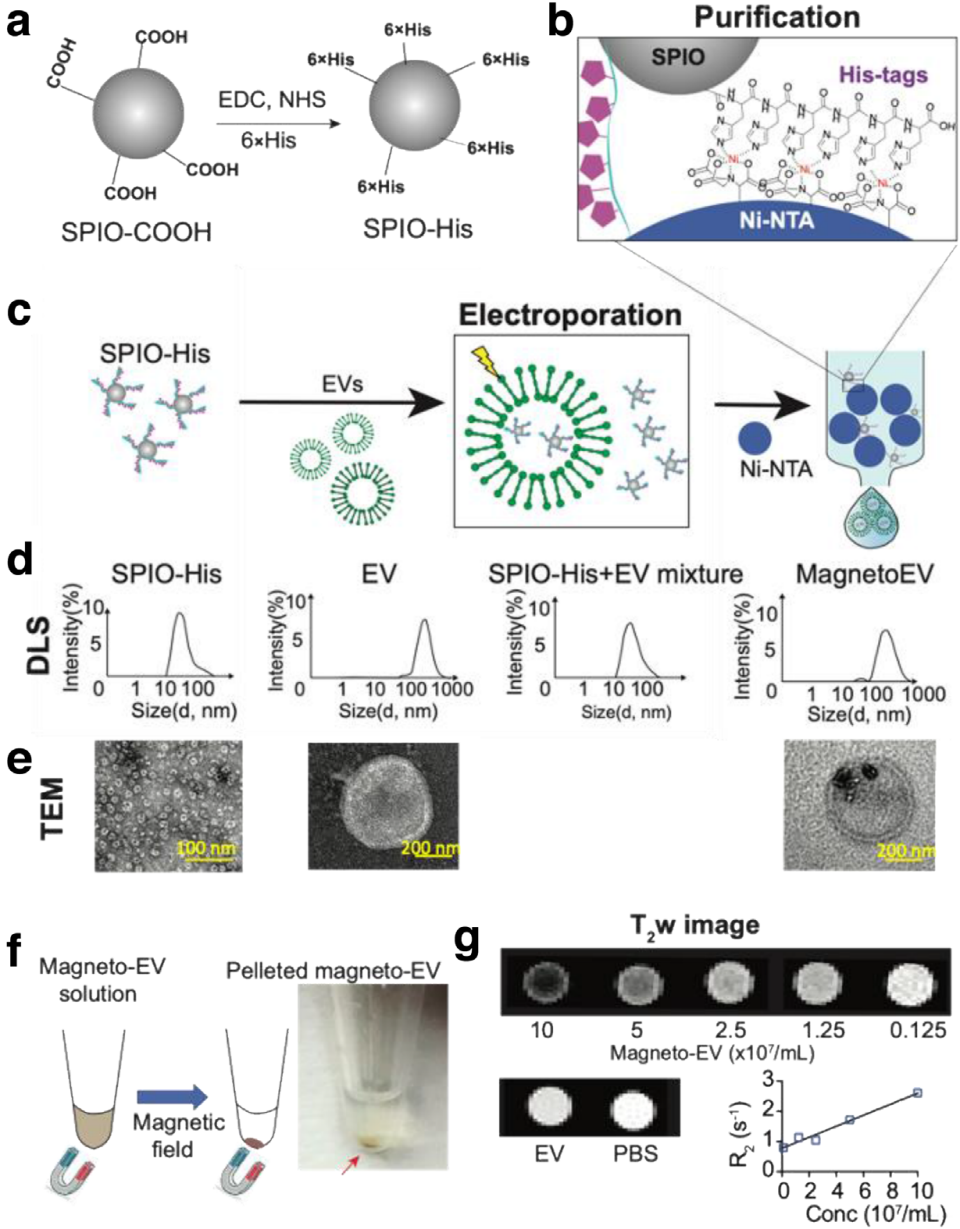
Preparation magneto‐EV and characterization of purified magneto‐EVs. (a) Schematic illustration of the preparation of SPIO his‐tag (SPIO‐His), by conjugating hexahistidine (6 × His‐tag) polypeptide to the carboxyl groups of SPIO particles using EDC (1‐ethyl‐3‐(3‐dimethylaminopropyl)‐carbodiimide), and NHS (sulfo‐N‐hydroxysuccinimide) chemistry. (b) As a result from the high affinity between the His‐peptide and nickel ion, the SPIO‐His particles bind to Ni^2+^ immobilized on beads (e.g. Ni‐NTA resins) for further purification. (c) Schematic illustration of the encapsulation of SPIO‐His into EVs by electroporation and subsequent purification by removing unencapsulated SPIO‐His from the elute using Ni‐NTA affinity chromatography. (d) Size distribution as measured by dynamic light scattering (DLS) for SPIO‐His, EVs, SPIO‐His/magneto‐EV/EV mixtures after electroporation, and the final purified elute, respectively. (e) TEM images of EV, SPIO‐His and EV‐SPIO, respectively. (f) Concentrating magneto‐EVs using a magnet. Eluted magneto‐EV solution was placed on a magnet overnight to pellet magneto‐EVs. The photograph shows the pelleted magneto‐EV at the bottom of a microcentrifuge tube. (g) T_2_‐weighted (T_2_w) images of magneto‐EVs at different concentrations, unlabelled EVs, and PBS. Mean R_2_ values of magneto‐EVs are plotted with respect to their concentration, from which the r_2_ (relaxivity) was estimated

To prepare ‘sticky’ magnetic nanoparticles, we first synthesized surface‐modified SPIO nanoparticles by conjugating them with hexa‐histidine peptides (6 × His) using the synthetic route shown in Figure 1a. With His‐tags on the surface, which was confirmed by Fourier‐transform infrared spectroscopy (Figure S1), SPIO nanoparticles can selectively bind to Ni^2+^ immobilized nitrilotriacetic acid (NTA) agarose resins (Ni‐NTA) (Figure 1b). This complex has a distinct colour (Figure S2), where the Ni‐NTA column eluted with SPIO‐COOH (no His tag) showed no colour change whereas the one eluted with SPIO‐His changed from light blue to brown.

The labelling and purification procedures of magneto‐EVs are illustrated in Figure 1c. In this study, we focused on iPSC derived EVs, which has been well characterized in our previous study (Liu et al., 2019). The synthesized SPIO‐His nanoparticles were first loaded into purified EVs by electroporation as described previously (Liu et al., 2019). The resulting solution containing a mixture of free SPIO‐His and magneto‐EVs was purified using a Ni‐NTA column. Quantitative analysis of iron content in the solution pre‐ and post‐elution revealed that a single‐time elution through the Ni‐NTA column was able to remove 97.4% of unincorporated SPIO‐His with minimal loss of EVs (∼5.4% as measured by nanoparticle tracking analysis). However, as we used a large amount of SPIOs (i.e. 50 μg, 2 mg ml^‐1^ in 25 μl) to load 1.1 × 10^11^ per ml EVs (50 μl) in a 75 μl system, a single elution can only remove 97.4% excess SPIOs, leaving 1.3 μg SPIOs in the solution (encapsulated SPIO = 55 ng). We therefore purified the solutions two more times to remove most of the excess SPIOs (Figure S2), with the residual SPIOs = 0.88 ng. The average size was measured using dynamic light scattering (DLS) to be 43.9 ± 16.5 nm, 248.2 ± 107.2 nm, 45.6 ± 15.9 nm, and 292.8 ± 113.0 nm, for SPIO‐His, EVs, unpurified magneto‐EVs, and purified magneto‐EVs, respectively (Figure 1d). The size distribution of magneto‐EVs closely resembled that of unlabelled EVs, indicating the efficient removal of unencapsulated SPIO‐His particles. Labelling and purification were verified by transmission electron microscopy (TEM) (Figure 1e and Figure S3), which showed no unencapsulated SPIO‐His particles in the purified solution, nor noticeable aggregated EVs. TEM demonstrated that many EVs contained multiple SPIO‐His particles. Moreover, the incorporation of magnetic particles allowed enrichment of magneto‐EVs using a magnetic force (Figure 1f). Compared to previously reported labelling method by which the SPIO‐ labelled EVs were harvested from parent cells that were pre‐incubated with SPIOs (Busato et al., 2016), our method provides a much higher labelling efficiency, that is 95.87% versus 19.32% of EVs were labelled with SPIOs among the totally collected EVs (Figure S4).

In vitro MRI confirmed the hypointense contrast of magneto‐EVs (Figure 1g). At 9.4 T and 37 °C, the R_2_ enhancement was determined to be 1.83 s^–1^ per 10^8^ per ml EVs, which is equivalent to an r_2_ relaxivity of 1.1 × 10^10^ s^–1^ mM^–1^ per EV (10^8^ per ml EVs = 16.7 pM) or 659 s^–1^ mM^–1^ Fe. Using the r_2_ relaxivities of SPIOs (i.e. 1.82 s^–1^ per mg ml^‐1^), we estimated the iron content in EVs to be ∼1 ng per 10^8^ EV. From this, we estimated the in vivo detection limit to be approximately 8.76 × 10^7^ EVs per ml EVs kidney tissue (having an inherent R_2_ of 32.03 s^–1^ at 3T (Ittrich et al., 2007)), assuming a 5% MRI signal change.

**FIGURE 2:**
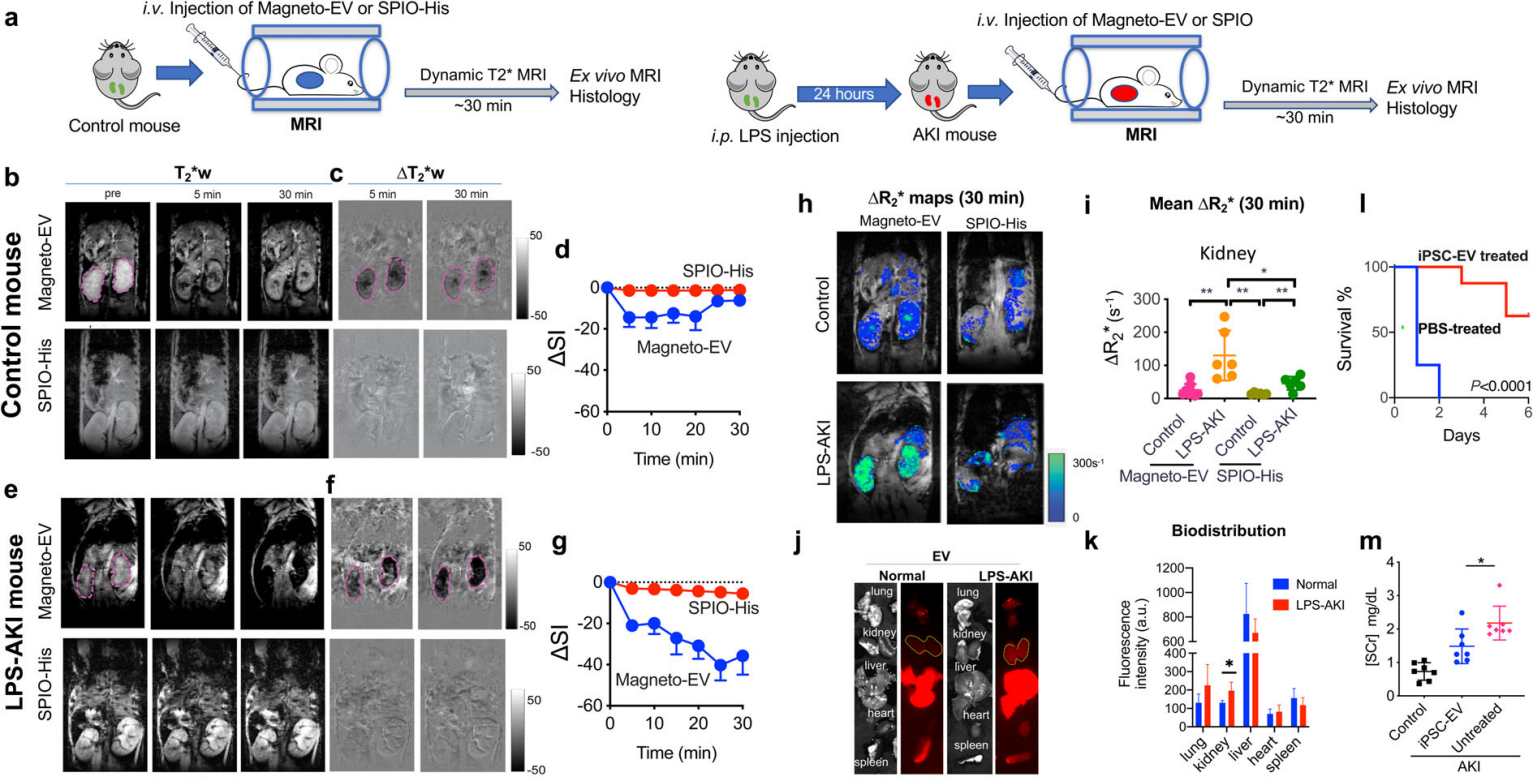
MRI detection of the uptake of magneto‐EV in control and injured kidneys in the LPS‐AKI model. (a) Timeline of preparation of animal model and injection of EVs. (b). T_2_*‐weighted (T_2_*w) images in a representative normal control mouse before and at 5 and 30 min after i.v. injection of magneto‐EVs and SPIO‐His, respectively. (c) Corresponding contrast enhancement maps, defined as ∆T_2_*w = T_2_*w (post)‐T_2_*w (pre). (d) Mean dynamic signal change in the control kidneys (*n* = 6). (e) T_2_*‐weighted (T_2_*w) images in a representative LPS‐AKI mouse before and at 5 and 30 min after the i.v. injection of magneto‐EVs and SPIO‐His, respectively. (f) Corresponding contrast enhancement maps. (g) Mean dynamic signal change in the injured kidneys of all three mice (totally six kidneys). (h) ΔR_2_*(defined as 1/T_E _× ln(SI^post^/SI^pre^)) maps at 30 min after injection of magneto‐EVs. (i) Quantitative comparison of mean ΔR_2_* values by in vivo MRI in the kidneys and liver at 30 min between different groups. (j) Representative bright‐field and fluorescence images of the kidneys, liver, spleen, heart and lung harvested from normal control (left) and LPS‐AKI mice (right) at 30 min after i.v. injection of iPSC‐EVs labelled with DiR. (k) Comparison of the mean fluorescence intensities among different organs in the control and LPS‐AKI groups* * * * ‐. (l) Survival curves of AKI mice treated with 2 × 10^9^ iPSC‐EVs and vehicle control (PBS). (m) Serum creatinine (SCr) levels at 24 h in the LPS‐AKI mice receiving iPSC‐EV or PBS (vehicle control), and normal mice without any treatment (negative control). EVs or vehicles were administered at the same time with LPS. * *P*< 0.05, **P< 0.01, unpaired two‐tailed Student t‐test.

We first assessed the distribution of magneto‐EVs in an lipopolysaccharides (LPS)‐induced acute kidney injury (AKI) model, a well‐established rodent model of AKI (He et al., 2020; Lv et al., 2020) associated with severe systemic inflammation and irreversible kidney damage within 24–48 h (Doi et al., 2009; Liu et al., 2018). For in vivo study, we used T_2_*‐weighted (T_2_*w) rather than T_2_‐wieghted MRI to detect SPIO‐containing magneto‐EVs with a higher sensitivity. It is because SPIO nanoparticles can strongly distort the local magnetic field and thereby cause a much quicker T_2_* decay of protons in nearby water molecules, which will appear as hypointense spots in T_2_*w MR images (Bulte et al., 2002; Huang et al., 2012). Our results show that magneto‐EVs generated sufficiently strong contrast for MRI detecting the dynamic uptake of magneto‐EVs following intravenous (i.v.) injection in uninjured control or LPS‐AKI mice (Supplementary Videos S1 and S2). As shown in Figure 2a, we used MRI to monitor and quantify the dynamic uptake of magneto‐EVs in the kidneys for 30 min after injection and compared with that of SPIO‐His particles. The 30‐min window was chosen because the blood half‐life of EVs has been reported to be 2–4 min only (Morishita et al., 2017).

Our results showed that injection of SPIO‐His nanoparticles caused negligible MRI signal change in the kidneys in both uninjured control mice and LPS‐AKI mice. In contrast, administration of magneto‐EVs resulted in distinctive dynamic MRI signal changes due to the homing of EVs. In uninjured control mice, we observed a rapid decrease in MRI signal intensity in the kidneys immediately after i.v. injection of magneto‐EVs, which then remained stable between 5 and 20 min and finally recovered to baseline at 20–30 min (Figure 2b‐d). In the LPS‐AKI mice contrast, we observed a substantially different kidney uptake pattern after injection of magneto‐EVs (Figure 2e‐g), with the MRI signal continuing to decrease for ∼25 min when it reached a plateau, indicating a continuous EV uptake and accumulation in the injured kidneys. At 30 min after magneto‐EV injection, the injured kidneys had little remaining MRI signal, indicating a substantially high accumulation of magneto‐EVs.

The amount of magneto‐EVs or SPIO‐His particles accumulated in the kidney was then quantified by the changes in R_2_* contrast in the kidneys, defined as ΔR_2_* = 1/T_2_*(post)‐1/T_2_*(pre) = 1/T_E_ × ln (SI^post^/SI^pre^), a commonly used metric in SPIO‐enhanced MRI (Feng et al., 2013). Snapshot ΔR_2_* contrast enhancement maps and changes at 30 min after magneto‐EV injection are shown in Figure 2h. Strong ΔR_2_* contrast enhancement was seen in the injured kidneys compared to uninjured control kidneys, indicative of a higher amount of magneto‐EVs accumulated in the injured kidneys, whereas weaker ΔR_2_* contrast enhancement was seen in both groups of kidneys injected with SPIO‐His nanoparticles. A quantitative comparison of kidney ROI values revealed a significantly higher ΔR_2_* for the LPS‐AKI group compared to uninjured controls (130.1 vs. 24.69 s^–1^, *P *= 0.0024) (Figure 2i). There was also a significant difference between the LPS‐AKI mice injected with magneto‐EVs versus SPIO‐His particles (130.1 vs. 46.0 s^–1^, *P *= 0.0254). The ΔR_2_* in the LPS‐AKI group after SPIO‐His injection was also higher than that in the normal control group (46.0 vs. 14.3 s^–1^, *P *= 0.0035). In addition, we calculated the dynamic ΔR_2_* values in the kidney for each group (Figure S5A,B), confirming the differential kidney uptake dynamics for LPS‐AKI mice injected with magneto‐EVs versus all other groups. The substantially increased uptake in the injured kidneys was further confirmed by ex vivo fluorescence imaging of fluorescently labelled iPSC‐EVs (Figure 2j,k), whereas uptake in the kidney was increased by nearly 50% (radiant efficiency = 130.8 and 196.1, respectively, *P = *0.0345) in the injured kidneys. Interestingly, while the liver always took up the highest amount of EVs among all organs imaged, the uptake in the liver of LPS‐treated mice was found to be slightly reduced as compared to the normal control. It should be noted that the MRI sequence used to acquire images in Figure 2 is not sensitive to detecting magneto‐EV or SPIO‐His accumulation in the liver due to the fact that the liver's intrinsic T_2_* time is short (∼ 4.5 ms). In order to detect a small amount of magneto‐EVs in tissues of low intrinsic T_2_*, more sophisticated MRI methods such as ultrashort T_E_ (UTE) sequence can be used. As shown in Figure S6A,B, UTE MRI revealed ∼3 times higher EV uptake in the liver than in the kidneys (Figure S6B), in good agreement with the fluorescence imaging (∼3.4 times higher, Figure 2k).

We also compared the biodistribution of iPSC‐EVs with that of liposomes, a nanoparticle with similar lipid bilayer membrane structures and size to iPSC‐EVs. The results show that the uptake of liposomes in injured kidneys (after normalized by the liver uptake in the corresponding mouse) was significantly less than that of iPSC‐EVs (*P *= 0.0252, *n* = 3, Student's t‐test) (Figure S7), indicative of the outperformed injury‐targeted ability of iPSC‐EVs than non‐targeted nanoparticles. As shown in Figure 2j, biodistribution of iPSC‐EVs in the lungs and heart were also increased in the LPS‐AKI mice compared to the normal control.

The accumulation of iPSC‐derived EVs in the injury sites resulted in an observable therapeutic effect. LPS‐AKI model has a rapid progression of kidney injury and, without treatment, the animal loss at 24 and 48 h was 87.5 and 100%, respectively. Compared to vehicle control (PBS), a single dose of 2 × 10^9^ EVs right after LPS injection could significantly improve kidney function as measured by animal survival (Figure 2l, *P *< 0.0001) and serum creatinine levels (Figure 2 m). When magneto‐EVs were injected at 3 h after LPS injection, a moderate improvement of survival (*P *= 0.0302, *n* = 5, Figure S8) could be observed; when magneto‐EVs were injected at 24 h, no improvement of survival (*P *= 0.1454, *n* = 5, Figure S8) was observed.

**FIGURE 3:**
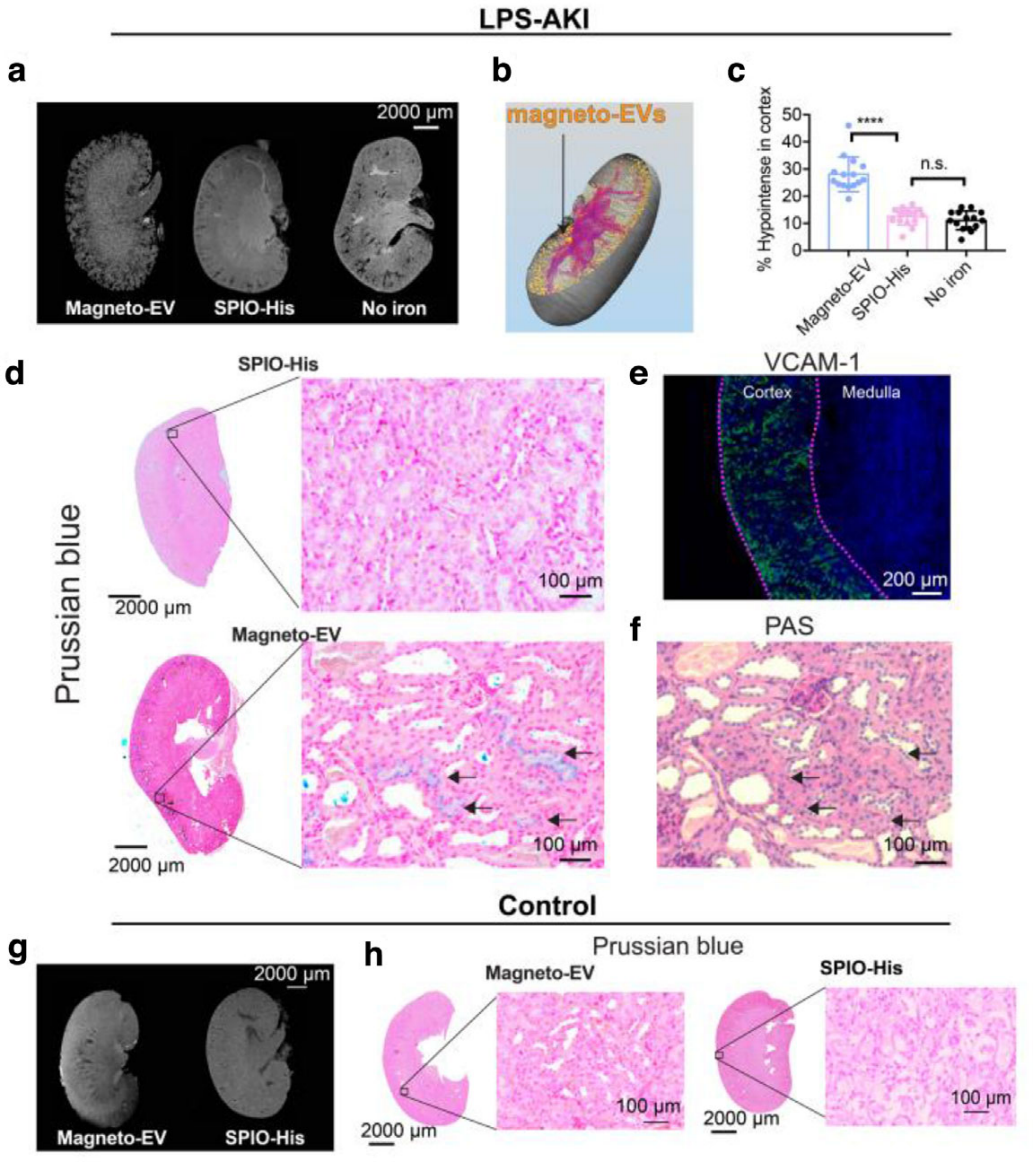
Distribution of magneto‐EVs in the injured kidney in the LPS‐AKI model. (a) Different distribution patterns of magneto‐EVs (left) and SPIO‐His (middle) in representative LPS‐AKI kidneys, as revealed by ex vivo high‐resolution MRI. Image of a representative non‐injected kidney on the right is shown as a control reference. (b) 3D reconstruction of a representative kidney showing the distribution of magneto‐EVs (gold‐coloured dots). Blood vessels are shown in purple colour (see also Supplementary Video 3). (c) Quantitative comparison of the relative hypointense areas (%) in the cortex of AKI mice injected with magneto‐EVs, SPIO‐His, or without injection (^****^ *P *< 0.0001, unpaired Student's t‐test, *n* = 15). (d) Prussian blue stains showing the distribution of magneto‐EVs and SPIO in the injured kidney (Left: whole kidney; Right: zoom‐in; Blue = SPIO; Red = nucleus). (e) VCAM‐1 staining of a representative kidney showing extensive inflammation (green) occurring in the cortex. Tissue was counterstained with DAPI (blue). (f) Periodic acid–Schiff (PAS) staining of the section corresponding to the Prussian blue stain on the left. (g) Distribution of magneto‐EVs (left) and SPIO‐His (right) in a representative normal control kidney. (h) Prussian blue stains showing the distribution of magneto‐EVs and SPIO‐His in uninjured kidney (Left: whole kidney; Right: zoom‐in; Blue = SPIO; Red = nucleus)

To study the spatial distribution of magneto‐EVs and naked SPIO‐His particles in kidneys in greater anatomic detail, we performed high resolution three‐dimensional (3D) ex vivo MRI on fixed kidney samples that were excised at 30 min after magneto‐EV or SPIO‐His injection. Figure 3a shows representative kidney T_2_*w images of LPS‐AKI mice injected with magneto‐EVs, SPIO‐His particles, and saline, respectively. Only the kidneys from mice receiving magneto‐EVs demonstrated a high number of hypointense spots and streaks dispersed throughout the renal cortex (Figure 3a, left). In contrast, kidneys of mice injected with SPIO‐His particles showed far fewer black spots (Figure 3a, middle), similar to the kidney without SPIO‐His injection (Figure 3a, right). The presence of black hypointensities in the last two groups may be due to the LPS‐related haemorrhage (H&E stain, Figure S9). 3D reconstruction of hypointense voxels revealed that magneto‐EVs distributed throughout the whole kidney, with preferential accumulation in the cortex (Figure 3b, full 3D visualization shown in Supplementary Video 3). Quantitative analysis showed that approximately 28.1% of the cortex of AKI mice injected with magneto‐EVs contained hypointensities (Figure 3c), significantly higher than those injected with SPIO‐His particles alone (12.5%, *P *< 0.0001) or saline (11%, *P *< 0.0001). Accumulation of magneto‐EVs in the cortex was confirmed by Prussian blue staining for iron (Figure 3d), where the tissue distribution of magneto‐EVs showed a good agreement with that seen on ex vivo MRI. Furthermore, immunostaining for VCAM‐1, a vascular inflammation marker, showed that LPS‐AKI kidneys exhibited higher VCAM‐1 expression in the cortex compared to the medulla (Figure 3e). Prussian blue‐positive iron also co‐localized with dilated proximal tubules as shown on Periodic acid–Schiff (PAS) staining (Figure 3f), with the tubular damage being a hallmark of the LPS‐AKI model (Zhang et al., 2017). In contrast, kidneys from control mice injected with either magneto‐EVs or SPIO‐His particles exhibited fewer hypointense areas, and those hypointense areas on T_2_*w images (Figure 3g) correlated well with the negative Prussian blue staining (Figure 3h). Of note, in vivo and ex vivo MR images may look different. For example, in vivo MRI indicated that, at 30 min post‐injection, a large amount of magneto‐EVs accumulated in the ureter of the control kidneys; but this contrast is absent in ex vivo images because the ureter structure and urine were lost during kidney dissection and processing. Also, in vivo MRI showed that magneto‐EVs dispersed throughout the LPS‐induced injured kidneys. In contrast, thanks to the higher signal‐to‐noise ratio (SNR) and spatial resolution, ex vivo MRI can not only reveal the widespread of magneto‐EVs but also the higher concentration of magneto‐EVs in the cortex than other regions.

To assess whether the accumulation of magneto‐EVs in the injured kidney and their subsequent beneficial effects are iPSCs specific, we also isolated EVs from FBS and injected them into LPS‐AKI mice using the same protocol as for iPSC‐EVs. The characterization of FBS‐EV is shown in Figure S10A. The ex vivo MRI results (Figure S10B) showed that a high quantity of FBS‐EVs accumulated in the cortex, suggesting our labelling and MRI detection approach can be applied to different types of (systemically‐injected) EV. The similar uptake patterns between FBS‐EVs and iPSC‐EVs may be attributed to the fact that both were derived from blood cells. However, despite their similar uptake pattern FBS‐EVs did not generate protection against the LPS‐induced kidney injury (Figure S10C, *P *= 0.1314). Further, serum creatinine (SCr) level in AKI mice received FBS‐EVs showed no improvement (Figures S10D, *P *= 0.9084). As revealed by proteomics analyses (Figure S11), iPSC‐EVs have numerous significantly altered proteins compared to serum‐derived EVs, with many of them associated with immune responses, wound healing, and hemostasis maintaining (Figure S12), which may explain why the EVs derived from iPSCs, but not those from serum, exhibited observable therapeutic effects on the injured renal cells,.

**FIGURE 4:**
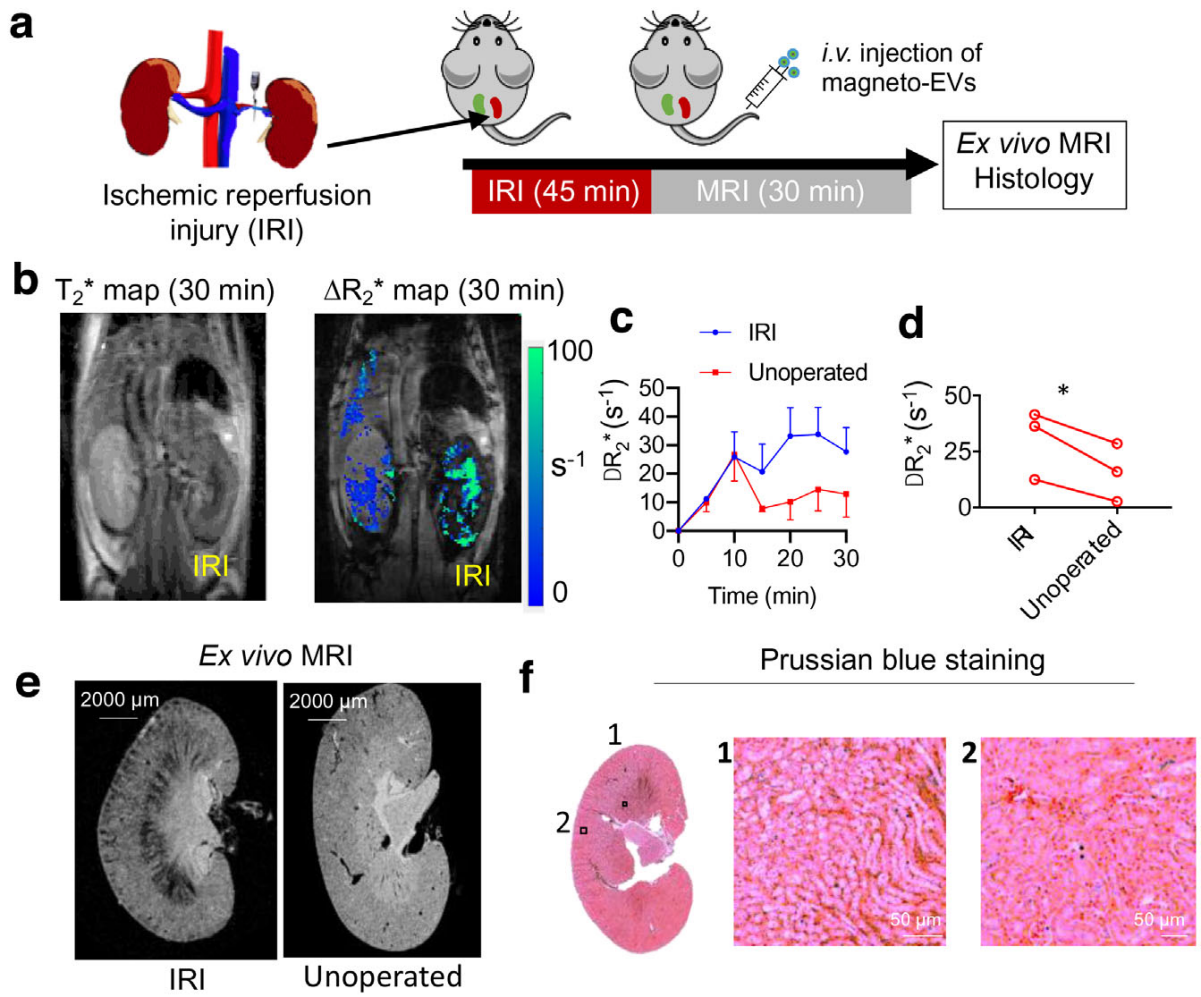
MRI tracking of i.v. administered magneto‐EVs in the IRI‐AKI model. (a) Schematic illustration of the experimental IRI‐AKI model and MRI acquisition. (b) Representative in vivo T_2_* image and ΔR_2_* map at 30 min after EV injection. (c) Dynamic ΔR_2_* MRI value changes in IRI and non‐operated control kidneys (*n* = 3 in each group). (d) Comparison of ΔR_2_* (30 min) values in IRI and control kidney for each mouse (^*^ *P *= 0.04, two‐tailed paired Student's t‐test, *n* = 3). (e) Ex vivo high‐resolution T_2_*w MR image of a representative IRI kidney and unoperated kidney. (f) Corresponding Prussian blue staining (Left: whole kidney; Right: zoom‐in; Blue = SPIO; Red = nucleus

We further investigated the homing ability of iPSC‐EVs to the injury sites in two other animal injury models using MRI. First, kidneys were subjected to a unilateral ischemia‐reperfusion injury (IRI) (Figure 4a). An acute injury in the right kidney was obtained by occluding the blood supply for 45 min, with the untreated left kidney as control. iPSC‐derived magneto‐EVs were administered i.v. at the same time when reperfusion started. As shown in Figures 4b,c, a higher EV uptake was observed in the injured kidneys but not the contralateral ones (∆R_2_* = 30.1 s^–1^ and 15.7 s^–1^, *P *= 0.04, two‐tailed paired Student's t‐test, *n* = 4). The pattern of magneto‐EVs homing to the injury site was distinct from the AKI mice induced by LPS injection. Both the ex vivo MRI (Figure 4e) and histology (Figure 4f) showed more magneto‐EVs accumulating in the medulla than the cortex, representing the difference in the primary site of injury between these two experimental models.

**FIGURE 5:**
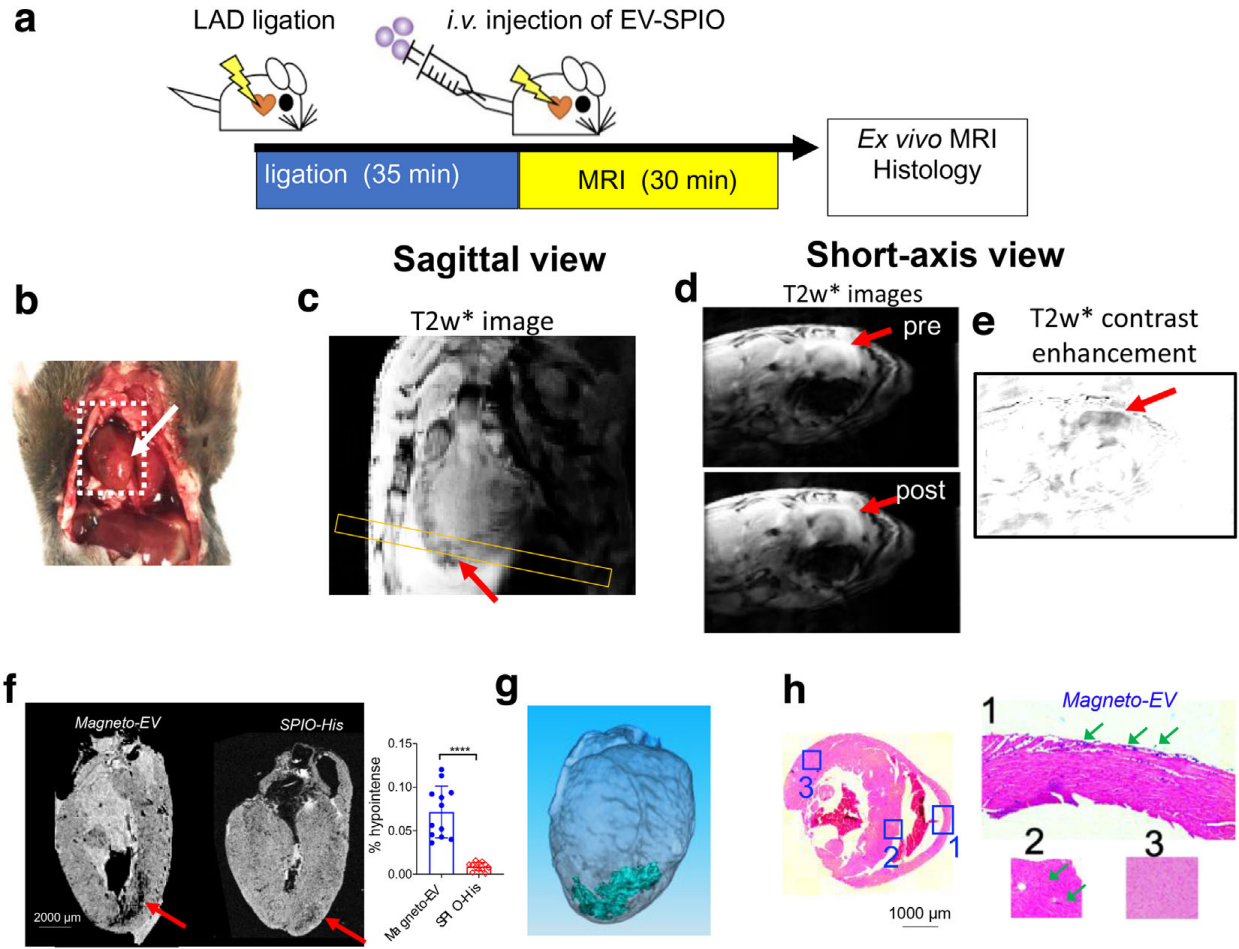
MRI tracking of magneto‐EV accumulation in the IR heart. (a) Schematic illustration of the experimental myocardial infarction mouse model and MRI acquisition. (b) Macrophotograph of the heart with the IR region (arrow). (c) Sagittal in vivo MR images of the heart. Yellow box indicates the slice position of the short‐axis view. Short‐axis pre‐ and post‐injection in vivo T_2_*w images (d) and enhancement maps, defined as ∆T_2_*w = T_2_*w (post)‐T_2_*w (pre) (e) showing hypointense areas in the injured region around the apex of the heart. (f) Ex vivo heart MR image showing higher accumulation of magneto‐EVs (red arrow) in injury region than that of SPIO‐His. The measured percentages of hypointense area in the myocardium of mice received magneto‐EVs or SPIO‐His are shown on the right. A total of 12 ex vivo MRI image slices were analysed for three mice in each group. ^****^ *P *< 0.0001, unpaired two‐tailed Student's t‐test. (g) 3D reconstruction showing the distribution of magneto‐EVs in the hearts. (h) Prussian blue staining of the injured heart (Left: whole heart of axial view; Right: zoom‐in of sections 1–3)

We then applied our technology to study the distribution of i.v.‐injected magneto‐EVs in a myocardial infarction (MI) mouse model, which was induced through the ligation of the left anterior descending (LAD) coronary artery for 35 min, followed by reperfusion (Pickard et al., 2017) (Figure 5a,b). The MRI results revealed that magneto‐EVs accumulated selectively at the injury sites pointed by red arrows in Figures 5c,d. While direct visualization of the MRI contrast caused by magneto‐EVs was only fairly conspicuous, the difference image (i.e. post ‐pre, Figure 5e) clearly showed the distribution of EVs in the injured myocardium, which is further confirmed by high resolution ex vivo MRI (Figure 5f). In comparison, magneto‐EV generated a significantly higher contrast in the injury hearts than SPIO‐His (Figure 5g). Prussian blue staining verified the accumulation of magneto‐EVs in the injured myocardium (Figure 5h).

## 4 DISCUSSION

The primary aim of our study was to develop a non‐invasive MRI tracking method for sensitive yet specific assessment of the homing of systemically‐injected EVs to injured renal and cardiac tissues in living subjects. Although there are several precedent studies elaborating on MRI detection of locally injected EVs (Busato et al., 2016; Hu et al., 2015), to the best of our knowledge, no study has been reported for tracking systemically administered EVs. In order to optimize the use of EVs as therapeutic agents, much needs to be learned about their pharmacokinetics, delivery and uptake efficiency as related to their dose, injection route, and cell origin. Using the new labelling strategy developed in the present study, we were able to prepare magneto‐EVs with sufficiently strong MRI signals that allowed us to dynamically and quantitatively track the delivery of intravenously injected, human iPSC‐derived EVs to the injured kidney and heart in different animal models.

The accurate detection of EVs in vivo is determined in large part by the quality of magneto‐EVs. The preparation of high‐quality magneto‐EVs requires not only the efficient labelling of EVs with magnetic particles to ensure a sufficient sensitivity for in vivo detection but also subsequent purification to ensure the specificity of MRI detection free of the interference of unencapsulated magnetic particles. While SPIOs have been used for cell labelling successfully (Baglio et al., 2012; Liu et al., 2018), it is much more challenging to achieve adequate MRI signal for EVs, whose volume are approximately five orders of magnitude (1.25 × 10^5^ times) smaller than that of their parental cells (diameter ∼10 μm). Hence, there may not be enough MRI detectability if the SPIO‐labelled EVs are prepared by collecting EVs from the cells pre‐incubated with SPIOs (Busato et al., 2016). On the other hand, while direct labelling EVs with magnetic particles using methods such as electroporation (Hood et al., 2014; Hu et al., 2015) and receptor‐mediated loading (on EV surface) (Qi et al., 2016) can provide a high labelling efficiency, the purification procedure is also challenging. Conventional methods are incapable of producing labelled EVs in high purity (especially for a large‐scale production) such that unencapsulated SPIOs are removed completely even using sophisticated and time consuming (i.e. 2 h (Hood et al., 2014)) purification steps.

To overcome these challenges, we developed an unprecedented approach to label EVs usingsurface‐modified, ‘sticky’ SPIOs. By the ability of His‐tags to bind with Ni‐NTA columns, the purification procedure becomes simple, less equipment‐intensive, and much faster (∼minutes), providing an efficient way to prepare magneto‐EVs at a high yield and, potentially, large scale. We used small commercially available SPIO nanoparticles with a 5 nm core size (∼46 nm hydrodynamic size) to maximize the encapsulation rate. As the purification step become very easy, we were able to use an extremely high concentration of SPIO (i.e. 0.67 mg ml^‐1^) during electroporation to enhance the labelling efficiency. In comparison, only 0.25 μg ml^‐1^ was used previously (Hood et al., 2014). The TEM results showed that the majority of EVs were successfully loaded with SPIOs, ensuring sufficient detection sensitivity. Finally, the electroporation conditions were optimized to ensure the variability of EVs, as evidenced by the comparison of size distribution and quality of EVs before and after electroporation‐purification as measured by DLS, and only a small portion (∼5%) of EVs was lost during the purification steps as measured by nanoparticle tracking analysis. The clinical application of EVs as therapeutics or drug carriers requires producing EVs at a large scale and, hence, scalability is an essential characteristic for any new EV technology. Our labelling method employs only an electroporator for SPIO‐loading and NTA columns for purification. Both instruments are scalable to a much larger volume, and the operation is time‐ and labour‐efficient. While EV isolation steps may cause substantial loss (e.g. estimated to be 44% in our study), there is a small loss in our labelling steps, making the overall yield still satisfactorily high. As a result, our approach has a good balance of purity against yield, making it suitable for future large‐scale applications. Of note, while demonstrated using Hist‐tags, our approach can be easily tailored to other ligand‐binding systems. Hence, our platform technology provides a simple yet efficient way for preparing highly purified magnetically labelled EVs.

The resulting magneto‐EVs have both high purity and sufficient MRI sensitivity, hence allowing accurate detection and measurement of the spatiotemporal quantity and distribution of systemically administrated EVs, without concerns of false positives caused by non‐encapsulated SPIOs. The estimated MRI detection limit is approximately 8.76 × 10^7^ EVs per ml by our in vitro data. With such a sufficient detectability, we were able to assess the uptake and distribution of iPSC‐derived EVs in the injured kidneys and hearts. In our study, we chose a dose from the dose range that were reported previously for treating AKI in murine models (i.e. 10^6^ to 10^10^ EVs per mouse (Choi et al., 2015; Ranghino et al., 2017; Viñas et al., 2016)). The dose used in our study (i.e. 2 × 10^9^ EVs or ∼3.4 μg proteins per mouse) is even lower than those used for SPECT (i.e. 29–64 μg^15,19^), optical imaging (i.e. 2.5–200 μg (Antes et al., 2018; Grange et al., 2014; Wiklander et al., 2015)), and CT (i.e. 2.8 × 10^9^ particles (Betzer et al., 2017)), as well as extracellular vesicle membrane‐coated nanoparticles for MRI (i.e. 3 × 10^11^ particles (Jc Bose et al., 2018)). It should be noted that the protein/particle ratio of 1.7 μg/10^9^ EVs was used only for the comparison purpose rather than as an absolute measure, as the particle to protein ratios in the literature vary greatly, ranging from 1 μg/10^10^ EVs for theoretically ‘pure’ EVs (Davidson et al., 2017) to 1 μg/10^8^ EVs (Pi et al., 2018). There are no previous MRI studies to detect intravenously injected EVs. The minimal amount of locally injected EVs to produce a reliable MRI signal were estimated to be 25 μg (Busato et al., 2016) to 50 μg (Hu et al., 2015). Compared to previous imaging methods, our technology provides a high sensitivity that permits MRI tracking of EVs in vivo.

Using the MRI signal of the magneto‐EVs, we were able to detect the dynamic uptake of EVs in the tissues of interest in different disease models non‐invasively. In the LPS‐AKI mice, both MRI and ex vivo fluorescence imaging revealed that magneto‐EVs accumulated in the injured kidney at a much higher level than in uninjured controls, which is consistent with a previously reported study on the increased MSC‐EV uptake in AKI mice using optical imaging (Grange et al., 2014). The distribution of EVs colocalized well with the injured proximal tubules in the cortex as previously reported for MSC‐derived EVs in a cisplatin‐induced AKI model (Zhou et al., 2013). While LPS‐induced septic shock may induce multi‐organ damage, our present study focused on the effect of EVs on the injured kidney as AKI is the major cause of sepsis associated mortality (Mårtensson & Bellomo, 2015). The accumulation of EVs in the injured areas was further confirmed in an IRI kidney model, where iPSC‐derived EVs preferentially accumulated in the medulla, in good agreement with previous reports on MSCs (Ittrich et al., 2007; Zhang et al., 2015). In addition, the accumulation of EVs provided significant protective effects and improved survival in the lethal LPS‐AKI model. However, only iPSC‐derived EVs but not FBS‐EVs produced a protective effect and improved the survival of mice with injured kidneys, even the latter exhibited similar distribution patterns. This supports the premise that the therapeutic effects are attributed to the cargo that EVs inherit from their parent cells, which was consistent with the proteomic analysis.

As expected, both MRI and fluorescence imaging revealed a substantially increased uptake of iPSC‐EVs in the kidneys after injury, although the uptake in the liver remains the highest among all organs studied as shown by both ultrashort TE sequence (UTE) MRI (Figure S6) and fluorescence imaging (Figure 2j,k). Interestingly, our results acquired using an UTE sequence (TE/TR = 2/20 ms) revealed a 3 times higher uptake in the liver than in the kidneys (Figure S6), whereas FLASH‐based acquisition (TE/TR = 5.8/800 ms) showed marginally similar uptakes in the liver and kidneys in the same animal model (Figure 2). Our data indicated that caution has to be taken to quantitatively compare the uptake of EVs in different organs using MRI.MRI sequences with short TE times are more suited forquantifying a small amount of magneto‐EVs in organs with short intrinsic T2/T2* times such as the liver. Further studies and optimization of MRI methods are required to fully understand the underlying mechanism of differential accumulation patterns of iPSC‐EVs compared with EVs from other cell origins, and to develop iPSC‐EVs to be a new drug delivery platform with the capacity of high delivery efficacy. Indeed, the cell origin of EVs could dramatically change the biodistribution of systemically injected EVs (Choi & Lee, 2018; Morishita et al., 2017; Wiklander et al., 2015), which highlights the urgent need for a quantitative, noninvasive imaging tool to accurately track EVs in vivo.

While we did not carry out a systematic study on the circulating time of intravenously injected iPSC‐EVs, our dynamic MRI studies showed that the uptake in the liver reached the steady state quickly (i.e. < 5 min), consistent with previous reports showing the blood half‐life of EVs is approximately 2–4 min in mice (Charoenviriyakul et al., 2017). As such, it is unlikely for EVs to release SPIO in the bloodstream. Of note, the pharmacokinetics of labelled EVs may be slightly different from that of naïve EVs (Gangadaran et al., 2017; Peinado et al., 2012). However, the impact of labelling is expected to be much smaller than the effects caused by cell source, route of administration, particle size, and surface post‐modification (Wiklander et al., 2015). Another limitation of our study is the small sample size involved in the treatment experiments. While the number of animals used in the present study is adequate to conclude the preventive effect of iPSC‐EVs when injected at an early time points (co‐injection and 3h after the injection of LPS), it may not be enough for treatment carried out at later time points, for example 24 h after LPS injection, due to their smaller effect size. Among numerous factors that can affect the outcomes, we speculate two major reasons responsible for the failure when EVs are administered later for AKI: (1) AKI develops very fast and become irreversible at late time points, and (2) substantially reduced perfusion in the kidneys as a consequence of injury in the late time points, which would seriously hamper the delivery of EVs. Hence, future studies are warranted to comprehensively investigate the therapeutic potential of iPSC‐EVs on AKI by using a larger sample size. It is also important to further investigate the dose‐dependent therapeutic effect and MRI detectability at different dose levels to fully explore the potential of MRI‐guided EV therapies. Finally, comprehensive investigations of the correlation between amount of EVs in the targeted tissues quantified by MRI and therapeutic outcomes in different in vivo disease models are needed to confirm the utility of MRI guidance for developing effective EV‐based therapies.

In sum, we developed a platform technology for preparing magneto‐EVs suited for in vivo MRI tracking. High efficiency of labelling and purifying EVs was achieved using SPIO particles functionalized with His‐tags, while non‐encapsulated particles could be easily removed by Ni‐NTA column. The produced magneto‐EVs were free of non‐encapsulated SPIOs, allowing accurate detection and measurement of the spatiotemporal distribution of therapeutic EVs without interferences. In vivo studies showed that magneto‐EVs derived from either human iPSC or FBS were capable of providing sufficient MRI signals to quantify the uptake of the intravenously injected EVs in injured organs, including kidneys and heart, correlating well with the histological assessment and therapeutic outcome in the LPS‐AKI model. Our technology paves a new pathway to accomplish in vivo tracking of therapeutic EVs and for guiding their applications in EV‐based regenerative medicine and possibly EV‐based drug delivery.

## AUTHOR CONTRIBUTIONS

Drs. Guanshu Liu, Linzhao Cheng,and Zheng Han conceived the research. Drs. Guanshu Liu and Zheng Han participated in all experiments and wrote the manuscript. Drs. Yigang Pei and Yuguo Li participated in animal model construction and MRI scans. Dr. Senquan Liu and Zheng Ding prepared iPSC‐derived extracellular vesicles. Drs. Daqian Zhan and Shuli Xia prepared FBS‐derived extracellular vesicles. Drs. Tom Driedonks and Kenneth Witwer characterized extracellular vesicles. Drs. Robert Weiss, Peter van Zijl, Linzhao Cheng, and Jeff Bulte reviewed and revised the manuscript.

## Supporting information

Supporting information.

Supporting information.

Supporting information.

Supporting information.
