# Supplementary material for: Highly efficient magnetic labelling allows MRI tracking of the homing of stem cell‐derived extracellular vesicles following systemic delivery
Source: J Extracell Vesicles. 2021 Jan 15;10(3):e12054. doi: 10.1002/jev2.12054 (PMC7809601; doi:10.1002/jev2.12054)
Supplement: Supplementary file 1 — Supporting information. [file JEV2-10-e12054-s001.pdf]

Supplementary information

Highly efficient magnetic labeling allows MRI tracking of the homing of stem cell-derived extracellular vesicles following systemic delivery

Zheng Han[1,2&], Senquan Liu[3,4,5 &], Yigang Pei[1,6&], Zheng Ding[3], Yuguo Li[1,2], Xinge Wang[7], Daqian Zhan[8], Shuli Xia[8], Tom Driedonks[9], Kenneth Witwer[9], Robert G. Weiss[1,10], Peter C.M. van Zijl[1,2], Jeff W.M. Bulte[1,2,3], Linzhao Cheng[4, 5*] and Guanshu Liu[1,2*]

[1] Russell H. Morgan Department of Radiology, Johns Hopkins University School of Medicine, Baltimore, MD, USA.

[2] F.M. Kirby Research Center, Kennedy Krieger Institute, Baltimore, MD, USA.

[3] Cellular Imaging Section and Vascular Biology Program, Institute for Cell Engineering, Johns Hopkins University School of Medicine, Baltimore, MD, USA.

[4] Department of Medicine, Johns Hopkins University School of Medicine, Baltimore, MD, USA.

[5] Division of Life Sciences and Medicine, University of Science and Technology of China, Hefei, Anhui, 230027, China

[6] Department of Radiology, Xiangya Hospital, Central South University, Changsha, Hunan, China

[7] Department of Bioengineering, University of Illinois at Chicago, Chicago, IL, USA

[8] Department of Neurology, Hugo W. Moser Research Institute at Kennedy Krieger, Baltimore, MD, USA

[9] Department of Molecular and Comparative Pathobiology, Johns Hopkins University School of Medicine, Baltimore, MD, USA

[10] Division of Cardiology, Department of Medicine, Johns Hopkins University School of Medicine, Baltimore, MD, USA

*: corresponding authors (Guanshu Liu: guanshu@mri.jhu.edu ; Linzhao Chen: lcheng2@jhmi.edu)

[&]: These authors contributed equally to this work

Supplementary Methods

S1. Preparation of liposomes

S2. Labeling liposomes with DiR dye

S3. Western blot analysis of EV markers

Supplementary Data

Figure S1. Fourier-transform infrared spectra of SPIO-His and SPIO-COOH.

Figure S2. Photos of SPIO-His (left) and magneto EVs (right) before (eluent) and after (eluate) passing through a Ni-NTA column.

Figure S3. TEM images of magneto-EVs.

Figure S4. Illustration of the experimental setup to compare labeling efficiency between our proposed method and the previously reported method.

Figure S5. Dynamics of ΔR_2^* contrast change in the kidneys generated by magneto-EVs.

Figure S6. MRI of magneto-EV uptake in the liver.

Figure S7. Ex vivo fluorescence imaging to assess the biodistribution of DiR-labeled EVs and liposomes.

Figure S8. Survival of AKI mice treated with $2x10^9$ iPSC-EVs.

Figure S9. Hematoxylin-eosin (HE) stain of a representative section of LPS-AKI kidney.

Figure S10. Characterization, MRI tracking, and effect on AKI of FBS-EVs.

Figure S11. LFQ intensity-based heatmap of the top differentially expressed proteins between iPSC-EVs and plasma derived EVs using |LFQ log2 fold-change intensity| ≥ 5 as a threshold.

Figure S12. GO pathway enrichment analysis for top differentially expressed proteins between iPSC-EVs and plasma derived EVs.

Movie S1. Dynamic T_2^*w images of a normal mouse after injection of magneto-EVs.

Movie S2. Dynamic T_2^*w images of an LPS-AKI mouse after injection of magneto-EVs

Movie S3. 3D visualization of the distribution of magneto-EVs in an LPS-AKI kidney.

Supplementary Methods

S1. Preparation of liposomes

Liposomes (DPPC: cholesterol: DSPE-PEG-2000 =57:40:3) were formed by the lipid film hydration method as described previously[1]. In brief, lipid mixture was dissolved in chloroform at the concentration of 25 mg/mL, followed by being air-dried for 90 min and vacuum-dried for 30 min. The lipid film was hydrated with 2 mL PBS (pH 7.4) at 50 ^{o}C for 1.5 h with intermittent shaking. Then the solution was extruded sequentially with 400 nm and 200 nm polycarbonate membranes. The size and distribution (polydispersity index, PDI) were measured at room temperature by dynamic light scattering using a Nanosizer ZS90 (Malvern Instruments, Southborough, MA) and the concentration of liposomes (in terms of number per milliliter) was measured using nanoparticle tracking analysis (Zetaview, Particle Metrix, Germany) after dilution. The size of liposomes was 226 nm in diameter (PDI=0.152).

S2. Labeling liposomes with DiR dye

DiR-labeled liposomes were prepared using the same protocol as that for EVs. In brief, liposomes of the concentration of 1.1×10^{11} per milliliter were added with DiR dye (1,1'-Dioctadecyl-3,3,3',3'-Tetramethylindotricarbocyanine Iodide, ThermoFisher Scientific) to a final concentration of 1 μM. After incubation for 15 min with gentle shaking, the free DiR dye was removed by eluting through a Sephadex G-50 column.

S3. Western blot analysis of EV markers

The iPCS-EV, E8 medium, FBS-EV, and FBS (5x diluted in PBS) were mixed with 4x XT sample buffer (BioRad) under non-reducing conditions, boiled for 5 minutes at 100°C, and subjected to PAGE gel electrophoresis on a 4%-15% Criterion TGX Stain-Free Precast gel (7.5%

for ApoB100 detection) (Biorad). Proteins were transferred to a PVDF membrane using the iBlot2 system (ThermoFisher) run at program p0 (for ApoB100: 1 min at 20V, 4 min at 23V, 5 min at 25V). After 1h of blocking in 5% Blotting-Grade Blocker (BioRad) in PBS + 0.05% Tween-20 (PBS-T), blots were incubated overnight at 4°C with the following primary antibodies in blocking buffer: mouse-anti-CD63 (1:500, sc-365604, SantaCruz), mouse-anti-CD81 (1 : 500, sc-23962, SantaCruz), and goat-anti-ApoB100 (1 : 1000, 20A-G1b, Academy Bio-Medical). Blots were washed 3x with PBS-T and incubated for 1.5h at room temperature with secondary antibodies, goat-anti-mouse-IgG-HRP (H + L; LI-COR Biosciences, Cat #P/N 925–32,210, Lincoln, NE, USA), and mouse-anti-goat-IgG-HRP (sc-2354, SantaCruz), all diluted 1 : 10,000 in blocking buffer. After washing 3× with PBS-T and 2× with PBS, SuperSignal West Pico PLUS Chemiluminescent Substrate (Pierce) was used for detection on an iBright FL1000 (ThermoFisher) in chemiluminescence mode.

Supplementary Data

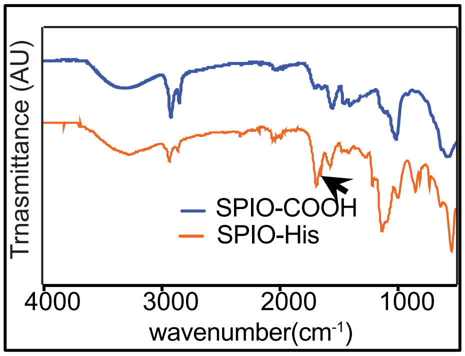

Figure S1. Fourier-transform infrared spectra of SPIO-His and SPIO-COOH. SPIO-His but not SPIO-COOH exhibited a characteristic peak of near 1680 cm^{-1}, confirming the formation of amide bonds between carboxyl group and the amine group of histidine peptide.

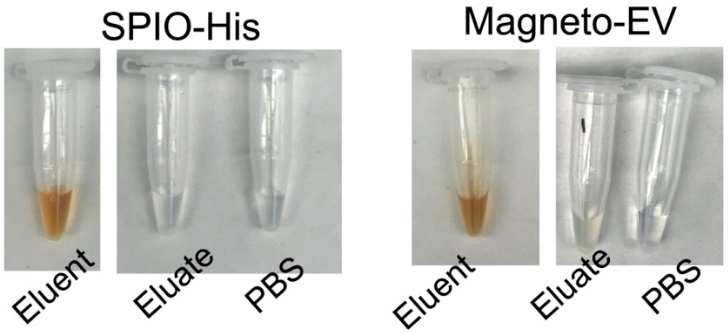

Figure S2. Photos of SPIO-His (left) and magneto EVs (right) before (eluent) and after (eluate) passing through a Ni-NTA column three times, with PBS solutions included as the control. Both SPIO-His and magneto-EVs eluents contain 0.67 mg/mL SPIOs. The eluate of SPIO-His after Ni-NTA column purification has no color (at least not perceptive), indicative of nearly complete removal of SPIO-His from the solution, whilst the eluate of magneto-EVs shows a light brownish color, indicating that a small amount of SPIOs exist inside EVs.

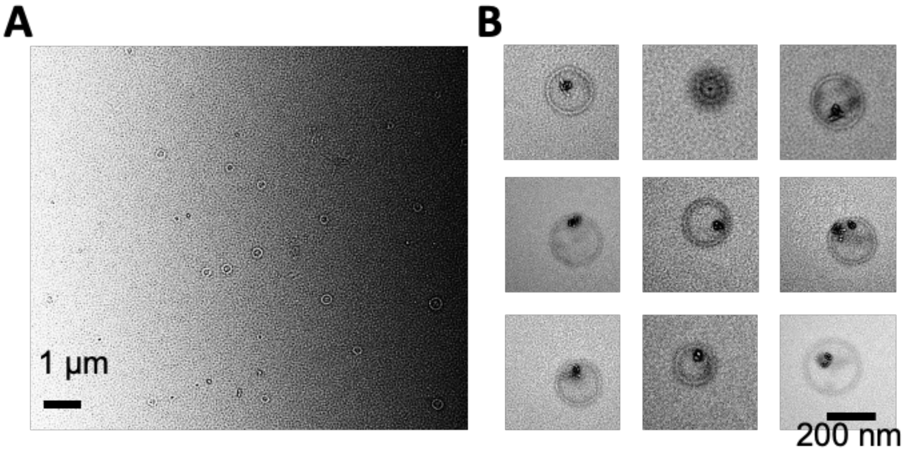

Figure S3. TEM images of magneto-EVs. A. A wide view TEM image of magneto-EVs. B. Zoom-in views of individual magneto-EVs in which SPIOs are clearly visible.

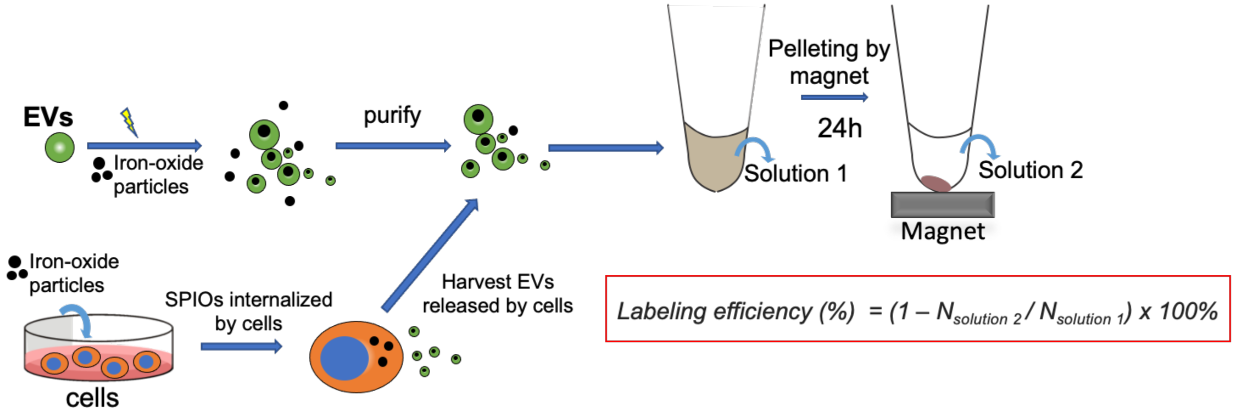

Figure S4. Illustration of the experimental setup to compare labeling efficiency between our proposed method and the previously reported method. Magneto-EVs were prepared using either our electroporation method or by harvesting EVs from parent cells pre-incubated with SPIOs for 24h. A Neodymium magnet fixed at the bottom of a microcentrifuge tube was used to separate magneto-EV from those without SPIO labeling in solution 1. Twenty-four hours later, the supernatant was collected (solution 2). The numbers of EVs in solution 1 and 2 were measured using nanoparticle tracking analysis (NTA), and the labeling efficiency was calculated as: labeling efficiency (%) $= (1 - N_{solution\ 2}/N_{solution\ 1})\mathrm{x}100\%$.

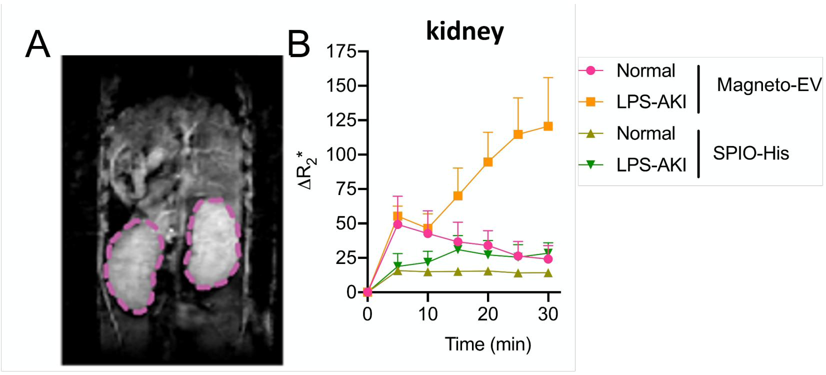

Figure S5. Dynamics of ΔR_2* contrast change in the kidneys generated by magneto-EVs. **A.** Coronal T_2*w image of a representative mouse showing the ROI selection of the kidneys. **B**. Dynamic changes of ΔR_2*, calculated by $1/T_E{}^* \times \ln(S^{post}/S^{pre})$, of kidney of normal and LPS-AKI mice after magneto-EV or SPIO-His injection.

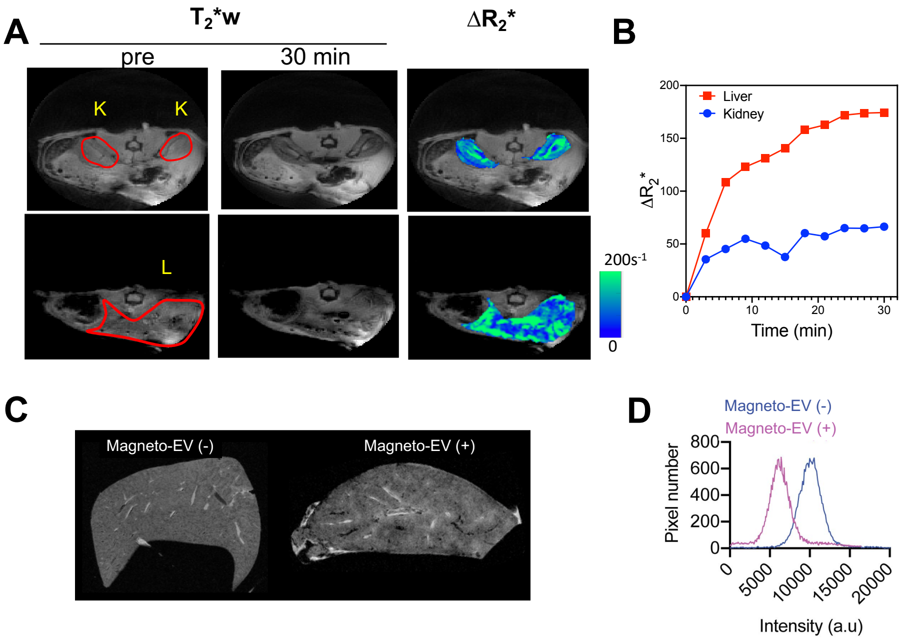

Figure S6. **MRI of magneto-EV uptake in the liver.** A. Axial UTE-MRI images containing either the kidneys (K, top panel) or liver (L, bottom panel) acquired prior to (pre) and at 30 min after magneto-EV injection. B. Dynamic changes of ΔR_2* of the kidneys and liver. C. Ex vivo MRI images of one liver samples harvested from LPS-AKI mice without (left) and with magneto-EV injection (right). D. Histogram of pixel intensities in the two livers shown in (C).

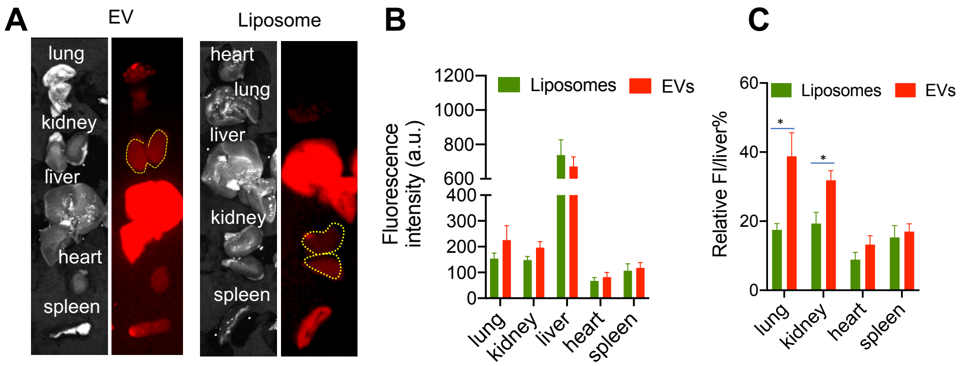

Figure S7. Ex vivo fluorescence imaging to assess biodistribution of DiR-labeled EVs and liposomes. A. Representative bright field and fluorescence images of lung, kidney, liver, heart and spleen of mice *i.v*. injected with DiR-labeled EV or liposomes. Organs were harvested at 30 min after injection. B. Quantitative analysis of the fluorescence intensities (without normalization) of different organs (n=4). C. Relative ratios of the mean fluorescence intensity in each organ to that in the liver. There was a significant difference between AKI and normal groups in the kidneys and lungs (*P*=0.0252 and 0.238 respectively, n=4, unpaired Student's t-test).

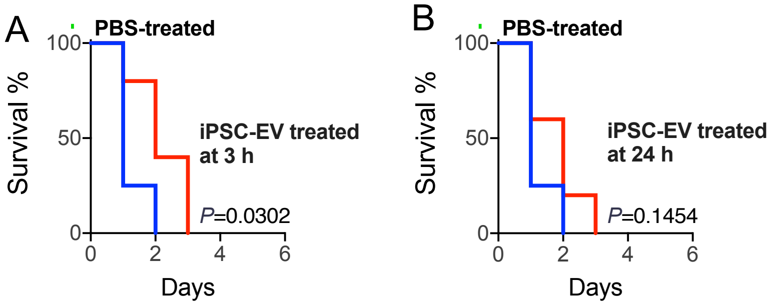

Figure S8. Survival of AKI mice treated with 2×10^9 iPSC-EVs at 3 (A) and 24 hours (B), as compared with the group of vehicle control (PBS). n=5 in each group.

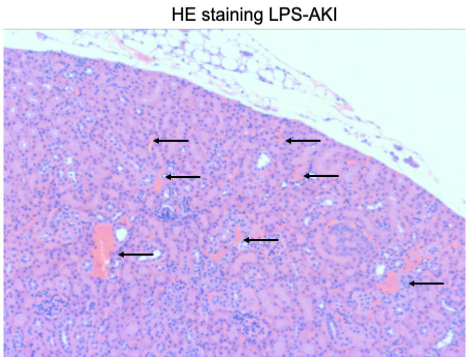

Figure S9. Hematoxylin-eosin (HE) stain of a representative section of LPS-AKI kidney, in which hemorrhages in the cortex of the kidney are indicated by black arrows.

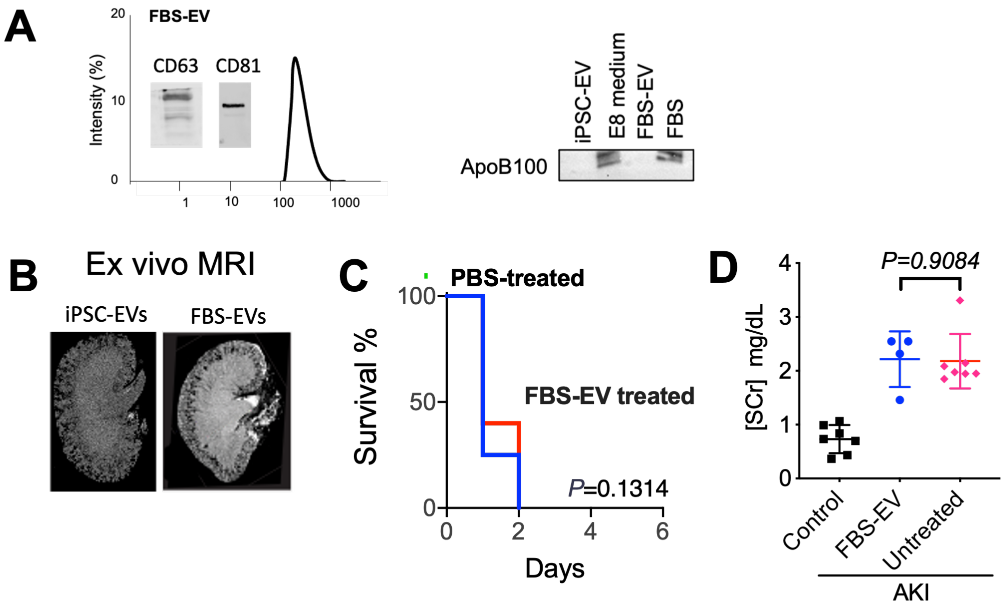

Figure S10. Characterization, MRI tracking, and therapeutic effect of FBS-EVs. (A) The characterization of FBS-EVs, including size (DLS measurement) and CD63, CD81, and ApoB100 expression (Western Blots). In the ApoB100 analyses, E8 medium and FBS were used as controls. The protein/particle ratio of FBS-EV was 12.5 μg/10^9 EV. **B.** *Ex vivo* MR images of a representative LPS-AKI kidney at 30 min after the injection 2×10^9 FBS-derived magneto-EVs. C. Survival of LPS-AKI mice co-injected with FBS-EV or PBS (vehicle control), respectively. D. Serum creatinine (SCr) levels at 24 hours in the LPS-AKI mice receiving FBS-EV or PBS (vehicle control), and normal mice without any treatment (negative control).

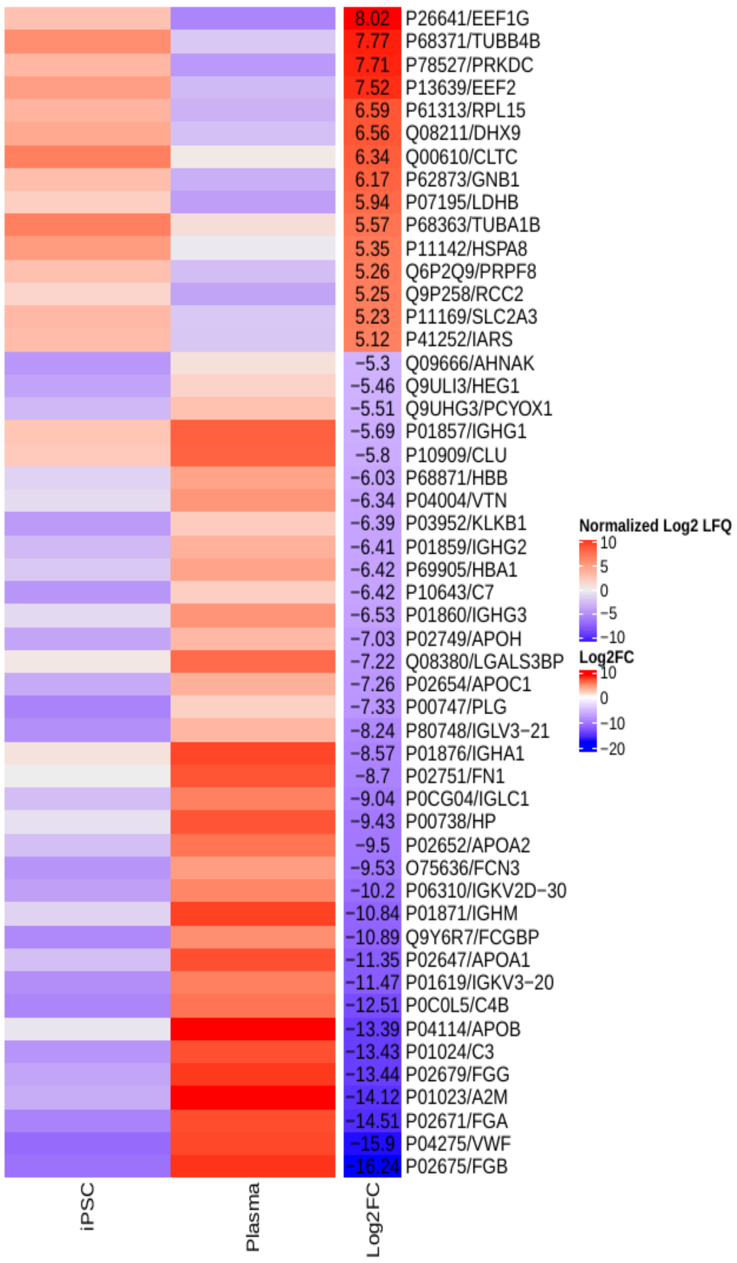

Figure S11. LFQ intensity-based heatmap of the top differentially expressed proteins between iPSC-EVs and plasma derived-EVs using |LFQ log2 fold-change intensity| ≥5 as a threshold. The first two columns of the heatmap are the normalized log2 LFQ intensity values from iPSC and plasma, respectively. The third column of the heatmap is the log2 fold-change comparison between iPSC-EVs and plasma derived-EVs, with red color representing higher expression in iPSC-EVs and blue color representing higher expression in plasma derived-EVs . The row name of the heatmap is formatted as “protein ID/corresponding gene symbol”.

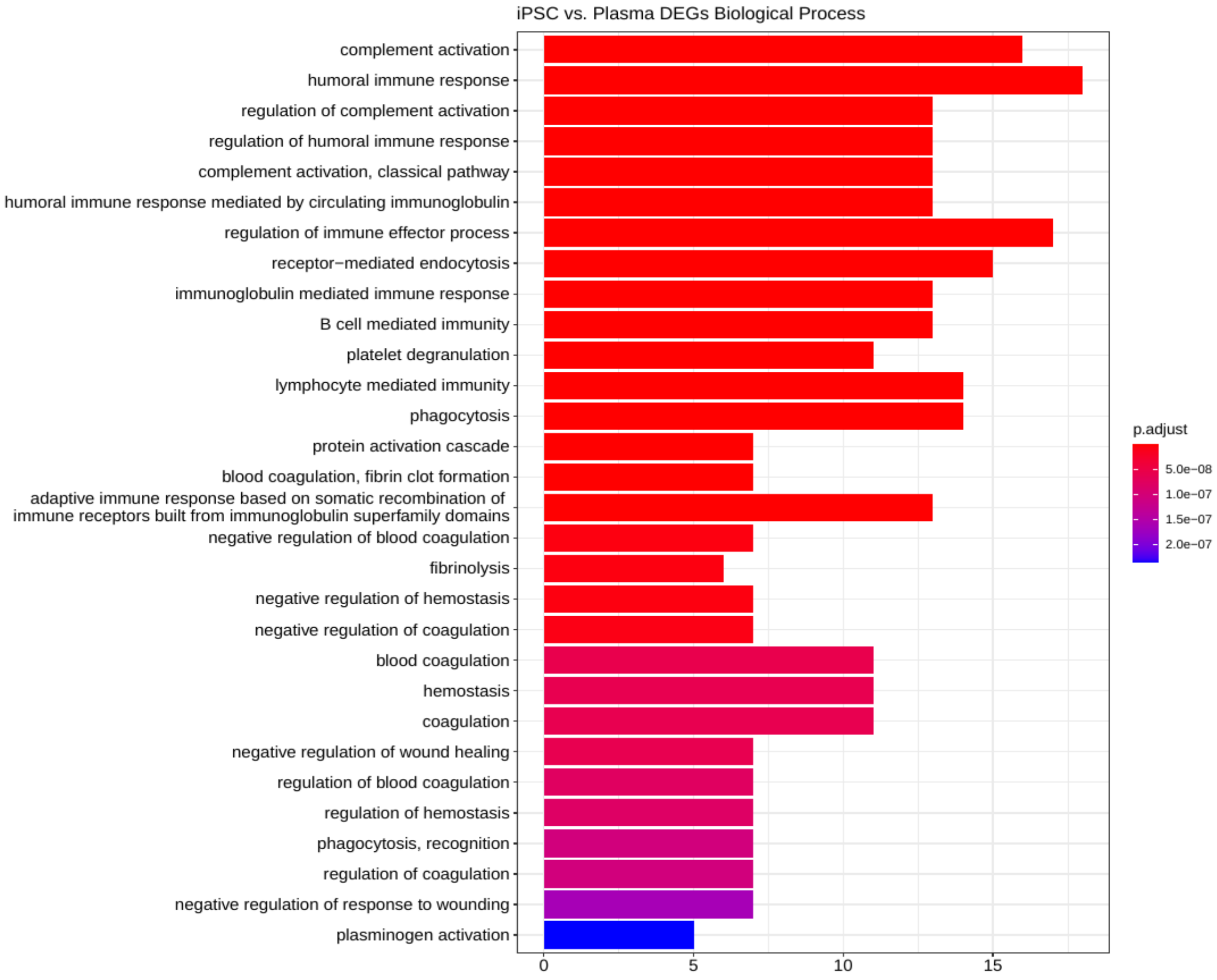

Figure S12. GO pathway enrichment analysis for top differentially expressed proteins between iPSC-EVs and plasma derived-EVs. p.adjust< 0.05 (multiple comparison adjusted p-values, using Benjamini-Hochberg procedure) was used as the threshold to select GO terms. Ontology.

Reference

1 Chen, Z. *et al*. CT and CEST MRI bimodal imaging of the intratumoral distribution of iodinated liposomes. *Quant Imaging Med Surg* **9**, 1579-1591, doi:10.21037/qims.2019.06.10 (2019).
